# A Role for p53 in the Adaptation to Glutamine Starvation through the Expression of SLC1A3

**DOI:** 10.1016/j.cmet.2018.07.005

**Published:** 2018-11-06

**Authors:** Mylène Tajan, Andreas K. Hock, Julianna Blagih, Neil A. Robertson, Christiaan F. Labuschagne, Flore Kruiswijk, Timothy J. Humpton, Peter D. Adams, Karen H. Vousden

**Affiliations:** 1 The Francis Crick Institute, 1 Midland Road, London NW1 1AT, UK; 2 Cancer Research UK Beatson Institute, Switchback Road, Glasgow G61 1BD, UK; 3 Institute of Cancer Sciences, University of Glasgow, Garscube Estate, Glasgow G61 1BD, UK; 4 Sanford Burnham Prebys Medical Discovery Institute, 10901 North Torrey Pines Road, La Jolla, CA 92037, USA

**Keywords:** p53, glutamine starvation, SLC1A3, aspartate metabolism

## Abstract

Numerous mechanisms to support cells under conditions of transient nutrient starvation have been described. Several functions of the tumor-suppressor protein p53 can contribute to the adaptation of cells to metabolic stress and help cancer cell survival under nutrient-limiting conditions. We show here that p53 promotes the expression of SLC1A3, an aspartate/glutamate transporter that allows the utilization of aspartate to support cells in the absence of extracellular glutamine. Under glutamine deprivation, SLC1A3 expression maintains electron transport chain and tricarboxylic acid cycle activity, promoting *de novo* glutamate, glutamine, and nucleotide synthesis to rescue cell viability. Tumor cells with high levels of SLC1A3 expression are resistant to glutamine starvation, and SLC1A3 depletion retards the growth of these cells *in vitro* and *in vivo*, suggesting a therapeutic potential for SLC1A3 inhibition.

## Highlights

• p53 promotes cancer cell proliferation and survival under glutamine starvation
• Aspartate metabolism supported by SLC1A3 is important under glutamine deprivation
• SLC1A3 expression is induced by p53
• Depletion of SLC1A3 impairs tumor growth *in vivo*

## Graphical Abstract

**Figure:**
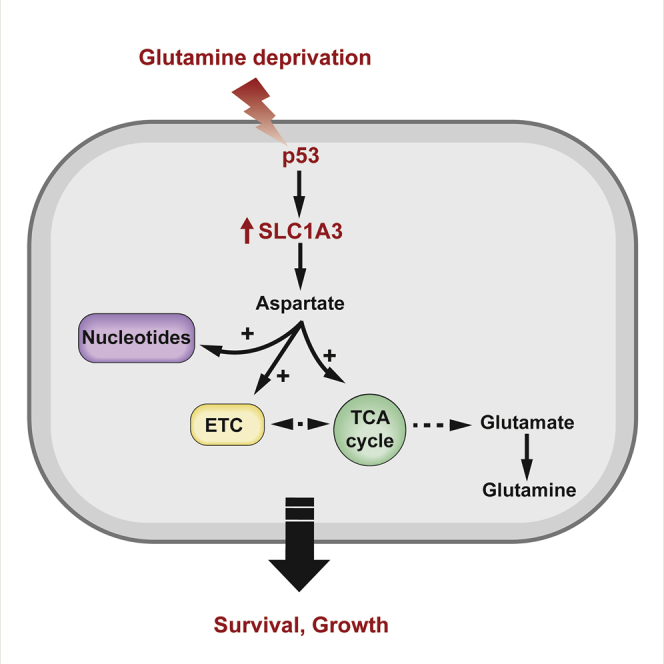

## Teaser

Tajan et al. show that the ability of cells to survive glutamine depletion depends on aspartate metabolism, which is supported by the aspartate/glutamate transporter SLC1A3. The tumor suppressor p53 induces SLC1A3 expression. Tumor cells with high SLC1A3 expression are resistant to glutamine starvation, and SLC1A3 depletion retards cell growth.

## Introduction

Cancer cells are frequently exposed to nutrient- and oxygen-limited environments, resulting from poor vascularization in the developing tumor mass, and there is a growing interest in understanding the metabolic plasticity that supports their survival and proliferation under these conditions. Glutamine is the most abundant amino acid in serum, and glutamine levels are often severely depleted in developing cancers (Kamphorst et al., 2015). Successful tumor development is therefore likely to depend on the ability of tumor cells to withstand glutamine depletion, and understanding the mechanisms involved may reveal new vulnerabilities for therapeutic targeting.

Glutamine contributes to nucleotide, amino acid, and protein synthesis, as well as glutathione production to support antioxidant defense (Altman et al., 2016). Glutamine can also be used to fuel the tricarboxylic acid (TCA) cycle, a pathway that depends on glutaminase (GLS) to catalyze the production of glutamate from glutamine. Several oncogenes such as Myc and KRas have been shown to alter glutamine metabolism leading to glutamine dependence, although the outcome can be strongly tissue and context dependent and some cancer cells produce glutamine from glutamate through a reaction depending on glutamine synthetase (GS) (Tardito et al., 2015). Intriguingly, asparagine has been shown to rescue death in response to glutamine starvation in glutamine-dependent cells (Zhang et al., 2014).

The *TP53* gene is frequently mutated in a wide range of different human cancers, with alterations or loss of p53 function detected in most epithelial malignancies (Vousden and Prives, 2009). As a transcription factor, p53 regulates the expression of a large number of genes that help to mediate the pleiotropic p53 responses. Wild-type p53 can inhibit proliferation or drive cell death, but can also help cells survive and repair genotoxic damage, both by promoting a transient cell-cycle arrest and through induction of DNA repair pathways. p53 activity is induced in response to serine or glutamine starvation (Maddocks et al., 2013, Reid et al., 2013) and the retention of wild-type p53 in cancer cells can help cells adapt to nutrient starvation through numerous mechanisms. These include the induction of a proliferative arrest to reduce metabolic demand, balancing pathways for energy production, limitation of oxidative stress, and regulation of genes that control specific metabolic pathways such as fatty acid oxidation (FAO) (Kruiswijk et al., 2015).

Here we identify SLC1A3 as a key mediator of p53’s ability to support cell survival and proliferation in the absence of glutamine. Cells expressing SLC1A3 maintain electron transport chain (ETC) and TCA activity, and the ability to synthesize glutamate, glutamine, and nucleotides, consistent with a previously described function of SLC1A3 in the transport of aspartate across the plasma and/or mitochondrial membranes. This activity allows for the utilization of aspartate, rendering cells capable of withstanding withdrawal of extracellular glutamine.

## Results

### Glutamine Starvation Activates a Protective p53 Response

**Figure 1:**
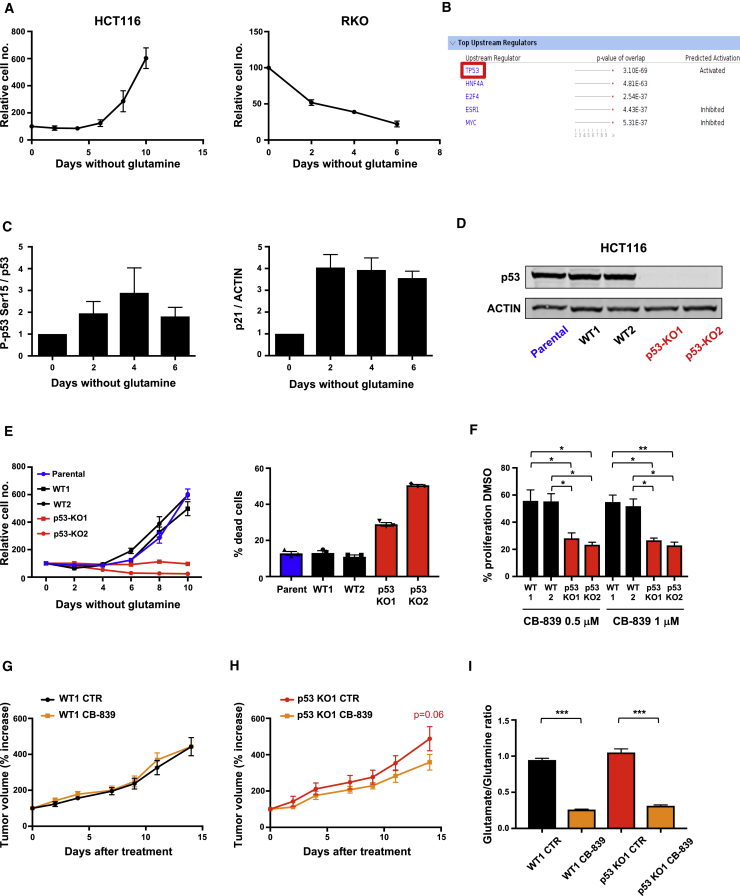
Glutamine Starvation Induces p53 Activation to Promote Survival and Proliferation (A) HCT116 and RKO cells were grown in glutamine-free medium and total cell numbers were counted every 2 days. Data are presented as means of triplicate wells ± SEM. (B) Ingenuity Pathway Analysis comparing HCT116 cells grown in complete medium versus those grown in equivalent medium lacking glutamine for 48 hr. (C) HCT116 cells were grown in glutamine-free medium for 6 days. Cell lysates were probed for phospho-p53(Ser15), total p53, p21, and ACTIN. Westerns blots from three independent experiments were quantified and data presented as mean ± SEM. (D) Western blot analysis shows p53 expression in HCT116 parental, wild-type (WT), and p53-null clones. (E) Growth assays of isogenic HCT116 cell lines under glutamine-free conditions (LHS). Data are presented as mean ± SEM of three wells from one representative experiment. Viability assays (RHS) of HCT116 isogenic cells after 6 days in glutamine-free medium. Graph represents the percentage of dead cells, and data are presented as mean of 3 wells ± SEM of one representative experiment. (F) HCT116 WT or p53-null clones were cultured for 3 days in complete medium with the glutaminase inhibitor CB-839 (0.5 μM or 1 μM). The graph shows the percentage growth compared with control condition (DMSO). Data are presented as mean ± SEM from three independent experiments (^∗^p < 0.05; ^∗∗^p < 0.01, paired two-tailed Student’s t test). (G) Tumor volumes of HCT116 WT1 xenografts dosed orally with vehicle (n = 5) or 200 mg/kg CB-839 (n = 5) twice daily for 14 days. The graph shows the percentage of increase in tumor volume from the initial tumor volume measured before treatment. Data are presented as mean ± SEM. (H) Tumor volumes of HCT116 p53-null clone 1 (KO1) xenografts dosed orally with vehicle (CTR) (n = 7) or 200 mg/kg CB-839 (n = 6) twice daily for 14 days. The graph shows the percentage of increase in tumor volume from the initial tumor volume measured before treatment. Data are presented as mean ± SEM (two-way ANOVA plus Bonferroni’s post hoc test). (I) Glutamate/glutamine ratio measured by LC-MS in tumor lysates from animals treated with vehicle (CTR) (n = 7 for WT1; n = 7 for p53 KO1) or CB-839 (n = 8 for WT1; n = 8 for p53 KO1), 4 hr after their final gavage and normalized to the tumor extract mass. Data are presented as mean ± SEM (^∗∗∗^p < 0.001, Mann-Whitney nonparametric test).

Previous studies have identified cell lines that differ in their sensitivity to glutamine starvation, as measured by induction of cell death (Cetinbas et al., 2016). A survey of a number of cancer cell lines reproduced this variation, showing that some cells (such as the colon cancer line HCT116) survived and continued to proliferate (albeit much more slowly) while others (e.g., the colon cancer line RKO) rapidly lost viability without glutamine (Figure 1A). To assess how cells that can adapt to glutamine starvation respond to this stress, we carried out RNA sequencing (RNA-seq) in wild-type p53-expressing HCT116 cells grown in medium containing all amino acids or without glutamine for 48 hr. Ingenuity Pathway Analysis (IPA) conducted on the CuffDiff differentially expressed genes (false discovery rate ≤ 0.05) revealed *TP53* as the most significantly enriched upstream regulator in the IPA analysis (p value of enrichment = 3.10 × 10^−69^); additionally, the directionality of the changes in expression of its downstream targets suggest that it is highly activated (activation *Z* score = 6.63) (Figure 1B). These results are consistent with a previous report showing activation of p53 in response to glutamine starvation in mouse embryo fibroblasts (Reid et al., 2013). The observed increase in p53 levels and phosphorylation, and expression of the p53 target gene p21 (Figure 1C), demonstrated the activation of a p53 response, which was transient, declining as the cells resumed proliferation. To establish the importance of p53 in this response, we generated independent p53-null HCT116 lines that failed to proliferate and showed decreased viability under glutamine starvation (Figures 1D and 1E). An alternative way to limit glutamine metabolism is by using a GLS inhibitor to block the production of glutamate from glutamine. Cells lacking p53 were more sensitive to CB-839, a GLS1 inhibitor (Gross et al., 2014), than wild-type p53-expressing cells, although the inhibitor slowed the proliferation of both cell types (Figure 1F) *in vitro*. *In vivo*, wild-type HCT116 xenografts were not affected by treatment of mice with CB-839 (Figure 1G), while the growth of p53-null HCT116 xenograft tumors was somewhat decreased in response to glutaminase inhibition (Figure 1H). This difference in sensitivity was not due to differential efficacy of the inhibitor, as there was a similar and substantial reduction in the ratio of glutamate to glutamine in both wild-type and p53-null tumors (Figure 1I).

**Figure 2:**
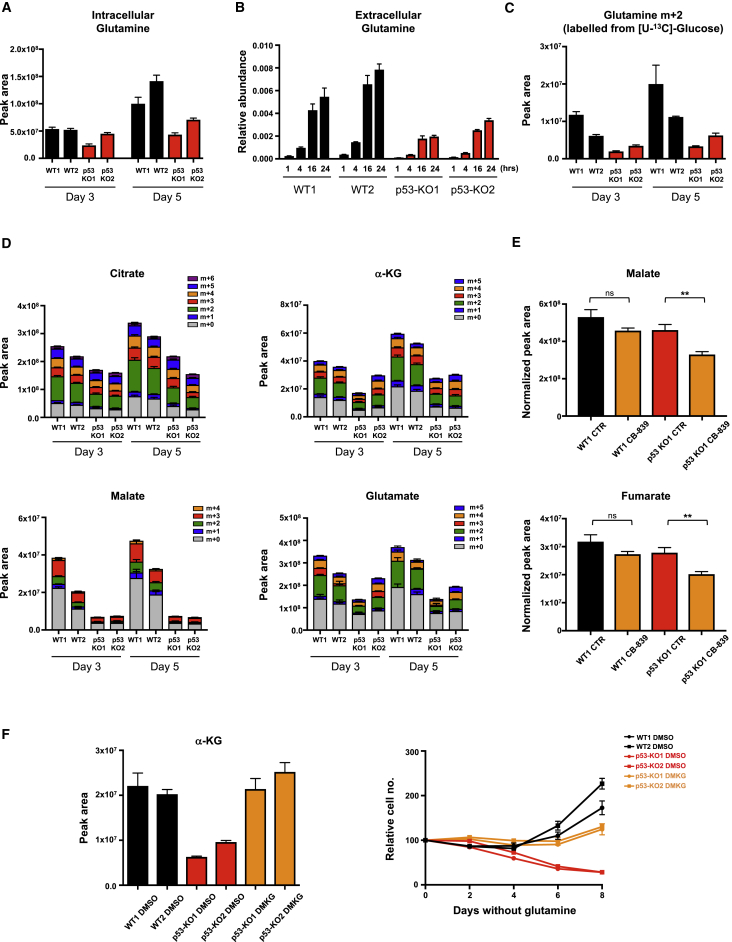
p53 Sustains TCA-Cycle Activity and *De Novo* Synthesis of Glutamate and Glutamine upon Glutamine Withdrawal (A) Intracellular glutamine levels in HCT116 isogenic cell lines grown for 3 or 5 days in glutamine-free medium (mean ± SEM of triplicate wells). (B) Cells were grown in glutamine-free medium for 4 days and extracellular glutamine levels quantified 1, 4, 16, and 24 hr after medium change, normalized to cell number (mean ± SEM of triplicate wells versus fresh medium). (C) HCT116 isogenic cell lines were cultured in glutamine-deficient medium for 3 or 5 days and stable isotopomer tracing analysis with [U-^13^C]glucose was performed. Metabolites were extracted and analyzed for glucose-derived isotopomer distribution of glutamine. Data are presented as mean ± SEM of triplicate wells. (D) TCA-cycle intermediates and glutamate analyzed as in (C). Data are presented as mean ± SEM of triplicate wells. (E) Metabolites were extracted from the tumors derived from HCT116 WT1 or p53 KO1 xenografts 4 hr after the final gavage with vehicle (CTR) (n = 7 for WT1; n = 7 for p53 KO1) or CB-839 (n = 8 for WT1; n = 8 for p53 KO1) and normalized to the tumor extract mass. Data are presented as mean ± SEM (^∗∗^p < 0.01, Mann-Whitney nonparametric test; ns, not significant). (F) Intracellular α-ketoglutarate levels in HCT116 WT and p53-null clones supplemented with or without dimethyl α-ketoglutarate (DMKG) under glutamine-free conditions (LHS). Data are presented as means ± SEM of triplicate wells. Proliferation (RHS) of WT and p53-null clones in the presence of DMKG. Data are presented as mean ± SEM of one representative experiment (averages of triplicate wells). See also Figure S1.

The activation of the cyclin-dependent kinase inhibitor p21 has been suggested to support cells under glutamine starvation by driving a cell-cycle arrest (Tran et al., 2017). However, we were interested in understanding whether p53 also contributes directly to the metabolic adaptation that allows proliferation in the absence of glutamine. Sestrin2, the product of a p53-responsive gene (*SESN2*), has been shown to help cells adapt to glutamine starvation by promoting FAO (Byun et al., 2017). Although we confirmed that *SESN2* transcription was strongly induced by glutamine starvation (Figure S1A), this was not dependent on p53 (Figure S1A). Analysis of intra- and extracellular glutamine levels showed an increase in both glutamine pools in wild-type p53 cells compared with the p53-null cells (Figures 2A and 2B). Furthermore, p53-null cells showed a decrease in flux from glucose into glutamine (Figure 2C), indicating that p53 expression results in an increased capacity to make glutamine *de novo*. GS catalyzes the condensation of glutamate and ammonia into glutamine, and wild-type p53 cells showed a strong stabilization of GS in response to glutamine starvation (Figure S1B), as previously reported (Nguyen et al., 2016). This response was somewhat blunted in the p53-null cells (Figure S1B). However, ectopic overexpression of GS in the p53-null cells was unable to rescue proliferation in the absence of glutamine (Figure S1C), suggesting that reduced GS expression was not the only defect in p53-null cells.

### p53-Null Cells Fail to Maintain TCA Cycle in Response to Glutamine Starvation

In many cancer cells glutamine is important in replenishing the TCA cycle, so we examined how p53 affected the accumulation of TCA-cycle intermediates in the absence of glutamine. Metabolomic analyses of cells fed uniformly ^13^C-labeled glucose showed that under glutamine starvation, p53-null cells contained lower levels of citrate, α-ketoglutarate (α-KG), and a very clear reduction in malate than their p53-expressing counterparts (Figure 2D). This was reflected by a decrease in labeling of these intermediates from glucose, and indicated that p53 helps to support TCA-cycle activity. Consistently, analysis of the HCT116 xenografts showed a reduction in TCA-cycle intermediates in response to glutaminase inhibition that was more pronounced in the p53-null tumors (Figures 2E and S1D). In glutamine-starved cells in culture, a reduction in glutamate was also detected (Figure 2D) that is likely to reflect, in part, the decrease in α-KG levels. As expected, based on previous observations (Wise et al., 2008, Zhang et al., 2014), addition of dimethyl α-KG (DMKG) and replenishment of intracellular α-KG levels greatly improved survival of glutamine-starved p53-null cells (Figure 2F), consistent with the suggestion that lack of α-KG is a cause of the lack of adaptation. However, careful titration of DMKG to restore levels of α-KG similar to those seen in p53 wild-type cells did not fully restore proliferation in these cells.

### Aspartate Metabolism Is Important under Glutamine Starvation and Is Defective in p53-Null Cells

**Figure 3:**
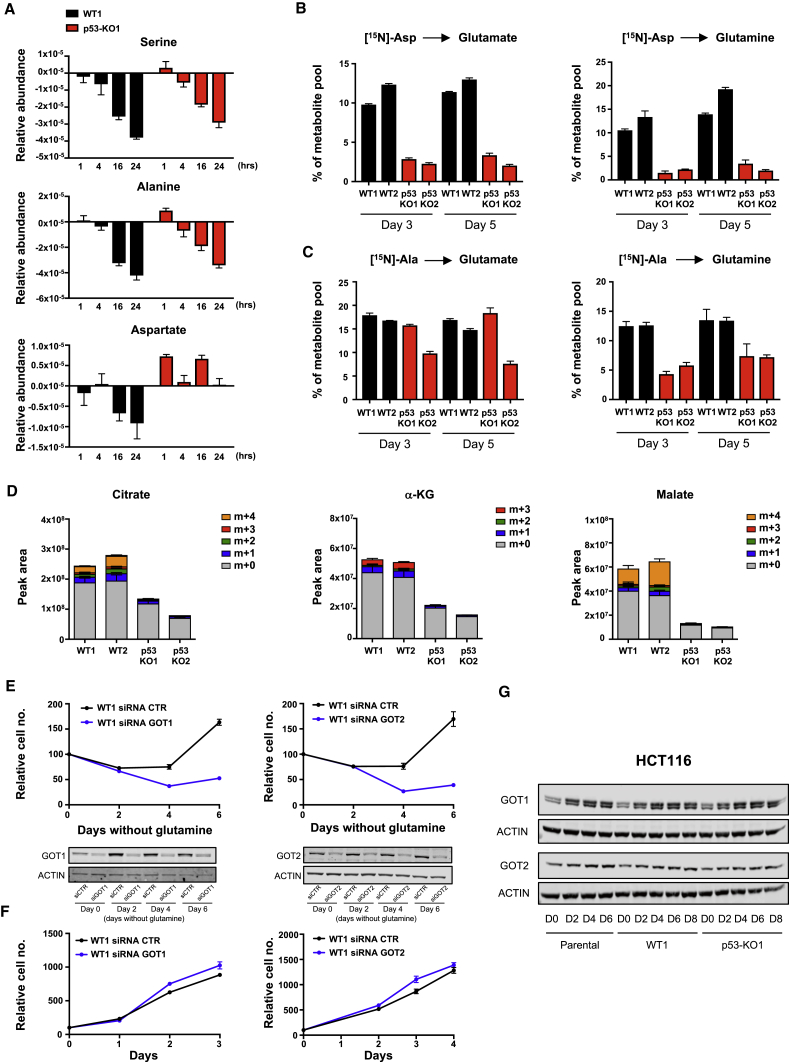
p53 Sustains Aspartate Metabolism under Glutamine Deprivation (A) HCT116 WT and KO clones were cultured in glutamine-free medium for 4 days. Extracellular serine, alanine, and aspartate, normalized to cell number, was quantified over 24 hr. Data are presented as mean ± SEM of one representative experiment (averages of triplicate wells). (B) Isotope tracing of [^15^N]aspartate into intracellular glutamate and glutamine. Metabolite percentages in glutamine-deprived HCT116 clones are represented as mean ± SEM of triplicate wells. (C) Isotope tracing of [U-^13^C, ^15^N]alanine into intracellular glutamate and glutamine. Metabolite percentages in glutamine-deprived HCT116 clones are presented as mean ± SEM of triplicate wells. (D) Stable isotopomer tracing analysis of [U-^13^C]aspartate incorporation into TCA-cycle intermediates in HCT116 isogenic cells 5 days after glutamine starvation. Data are presented as mean ± SEM of one representative experiment (averages of triplicate wells). (E) Proliferation of HCT116 WT cells transiently depleted of GOT1 and GOT2 using short interfering RNA (siRNA) and cultured in glutamine-free condition for 6 days. Data are presented as mean ± SEM of one representative experiment (averages of triplicate wells). The downregulation of these two enzymes was confirmed by western blot analysis (bottom panels). (F) Proliferation of HCT116 WT cells transiently depleted of GOT1 and GOT2 under fully fed conditions. Data are presented as mean ± SEM of one representative experiment (averages of triplicate wells). (G) Western blot analysis demonstrating the time course of GOT1 and GOT2 expression in HCT116 parental, WT1, and p53-KO1 clones grown in glutamine-free medium over 8 days. See also Figures S2 and S3.

We considered the possibility that the inability of α-KG to fully rescue growth of the p53-null cells reflects a defect in the acquisition of a nitrogen source, which is also required to produce glutamate and glutamine. Cells can produce glutamate directly from α-KG and ammonia (Spinelli et al., 2017) or from α-KG plus other amino acids such as alanine, serine, or aspartate. Both p53 wild-type and p53-null cells depleted the culture medium of alanine and serine under conditions of glutamine starvation, suggesting that access to a nitrogen source was not a critical difference between these two cell types (Figure 3A). Culture of wild-type p53 cells also led to a decrease in extracellular aspartate, although this was more modest than seen for alanine and serine (Figure 3A), reflecting a relatively inefficient aspartate transport in non-CNS cells (Birsoy et al., 2015). By contrast, the p53-null cells did not deplete extracellular aspartate (Figure 3A), and while nitrogen from ^15^N-labeled aspartate was detected in glutamate and glutamine in wild-type p53-expressing cells, this was greatly decreased in p53-null cells (Figure 3B). Previous studies have shown that *de novo* glutamine synthesis utilizes alanine (Tardito et al., 2015) and we also saw a substantial contribution of alanine-derived nitrogen to glutamate and glutamine in p53-expressing cells (Figure 3C). Interestingly, although the p53-null cells incorporate less alanine-derived nitrogen into glutamine, this effect is less pronounced than the failure to utilize aspartate. These results suggest that p53-null cells are defective in some aspect of aspartate metabolism. Aspartate plays a key role in nucleotide synthesis, and we detected a decrease in *de novo* synthesis of purines and pyrimidines (seen as a decrease in ADP, ATP, UDP, and UTP) from glucose in p53-null cells (Figure S2A). Nucleoside supplementation improved the proliferation of wild-type cells and supported survival of p53-null cells (Figure S2B) under glutamine starvation, suggesting that a defect in aspartate utilization into nucleotide synthesis is one factor leading to the death of the p53-null cells.

Further analysis of the fate of aspartate in these cells also identified aspartate-derived carbons in the TCA-cycle intermediates citrate, α-KG, and malate in the wild-type p53, but not p53-null cells (Figure 3D). Of note, the strongest difference was in the loss of the m+4 isotopomer of malate, which was predominant in the wild-type p53-expressing cell. While a reduction in m+4 malate could reflect the decrease in purine synthesis (which would generate m+4 fumarate), another source of this m+4 malate is by direct synthesis from aspartate-derived oxaloacetate rather than through the TCA cycle via α-KG. The utilization of aspartate for oxaloacetate and glutamate production requires the action of the cytosolic and mitochondrial transaminases, GOT1 or GOT2. Depletion of either GOT1 or GOT2 in wild-type p53-expressing cells severely impeded their ability to proliferate under glutamine starvation (Figure 3E), although there was no apparent contribution of either enzyme to cell growth under fully fed conditions (Figure 3F). Interestingly, glutamine depletion led to the upregulation of both GOT1 and (to a more modest extent) GOT2 protein expression (Figure 3G), with an increase in *GOT1* (but not *GOT2*) mRNA expression evident in the RNA-seq experiment (Figure S3A). However, this elevation of GOT1 and GOT2 expression was seen in both p53-expressing and p53-null cells (Figure 3G), indicating that a failure to induce GOT1 and GOT2 is not the crucial defect in p53-null cells. Expression of MDH1 and MDH2, the second enzymes involved in the conversion of aspartate to malate, was not obviously changed in response to glutamine starvation and was maintained in p53-null cells (Figure S3B). These results indicate that aspartate utilization becomes critical to allow adaptation to glutamine starvation, and that multiple aspects of aspartate metabolism are defective in p53-null cells.

### p53 Induces Expression of SLC1A3, which Sustains Cells under Glutamine Starvation

**Figure 4:**
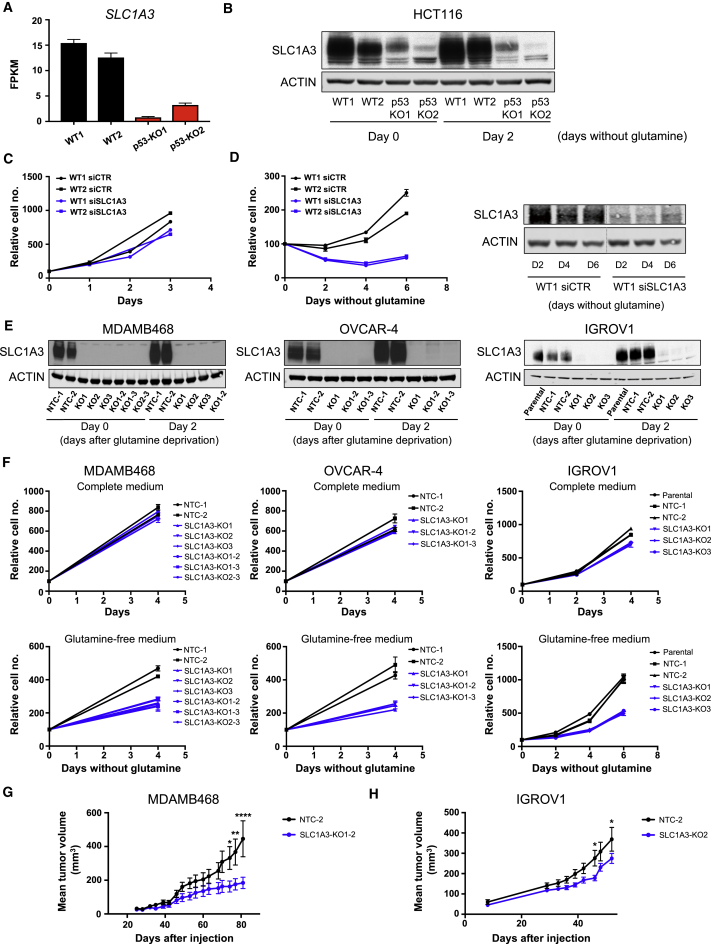
SLC1A3 Is a p53 Target that Sustains the Growth of Cancer Cell Lines under Glutamine Deprivation (A) *SLC1A3* transcriptional expression in HCT116 WT and p53-null clones grown for 2 days in glutamine-free medium. Data are presented as mean ± SEM (averages of triplicate wells). (B) Western blots show SLC1A3 expression in HCT116 WT and p53-null clones grown for 2 days in glutamine-free medium. Note that AGC2 and SLC1A3 (Figure S7D) were probed on the same membrane, so the same ACTIN western blot is shown here and in Figure S7D. (C and D) HCT116 WT cells were transiently depleted of SLC1A3 using siRNA, and proliferation assessed in complete medium (C) or in glutamine-free medium (D). Data are presented as mean ± SEM of one representative experiment (averages of triplicate wells). The downregulation of SLC1A3 was confirmed by western blot (D, right panel). Note that siCTR and siSLC1A3 samples were run on the same gel but intervening lanes have been removed (cut indicated by gray line). (E) MDA-MB-468, OVCAR-4, and IGROV1 cells infected with Cas9/SLC1A3 single guide RNA were cultured in glutamine-free medium for 2 days. Western blot shows efficient SLC1A3 depletion in Cas9/SLC1A3-infected cells. (F) Proliferation rates of cells cultured in complete medium (top panel) or in glutamine-free medium (bottom panel). Data are presented as mean ± SEM of one representative experiment (average of triplicate wells). (G) BALB/c nude mice were subcutaneously injected with MDA-MB-468 NTC cells (n = 6) or SLC1A3 KO cells (n = 8). Once the tumors were palpable, tumor volumes were measured twice a week by caliper measurement. Data are presented as mean ± SEM (^∗^p < 0.05, ^∗∗^p < 0.01, ^∗∗∗∗^p < 0.0001, two-way ANOVA plus Bonferroni’s post hoc test). (H) CD-1 nude mice were subcutaneously injected with IGROV1 NTC cells (n = 9) or SLC1A3 KO cells (n = 10). Once the tumors were palpable, tumor volumes were measured twice a week by caliper measurement. Data are presented as mean ± SEM (^∗^p < 0.05, two-way ANOVA plus Bonferroni’s post hoc test). See also Figures S4 and S5.

To understand how p53 affects aspartate metabolism, we looked for p53-dependent expression of genes associated with aspartate metabolism and identified the aspartate/glutamate transporter, SLC1A3 (EAAT1) (Figure 4A). While SLC1A3 was slightly induced in response to glutamine starvation in wild-type p53-expressing cells, a considerable reduction in expression of SLC1A3 was seen in the p53-null cells under both fed and starved conditions (Figure 4B). Depletion of p53 from the wild-type cells using small interfering RNA (siRNA) also led to a decrease in SLC1A3 expression, which was most evident in response to glutamine starvation (Figure S4A). We also noted p53-dependent *SLC1A3* expression in a published study (Miyamoto et al., 2017), while sequence analysis using the Broad Institute gene set expression analysis site identified *SLC1A3* as a potential transcriptional target of p53. Although depletion of SLC1A3 did not profoundly affect the growth of wild-type p53-expressing cells under fully fed conditions (Figure 4C), there was a clear defect in cell growth in response to glutamine starvation (Figure 4D) very similar to that seen in the p53-null cells (Figure 1E).

To determine the general role of SLC1A3 in the response to glutamine starvation, we examined a panel of cell lines derived from different cancer types. While most cell lines showed very low or undetectable levels of SLC1A3 protein expression (A2780, A549, U2OS, OVCAR-5, RT4, SiHa, and A375), others expressed high levels of SLC1A3 (MDA-MB-468, OVCAR-4, and IGROV1) (Figure S4B). Importantly, the levels of SLC1A3 seen in these three cell lines were substantially higher than that detected in wild-type p53 HCT116 cells (Figure S4D). Interestingly, there was a strong correlation between the ability of these cells to grow in the absence of glutamine and expression of SLC1A3 (Figures S4B and S4C). Glutamine depletion induced the expression of SLC1A3 in each of these cell lines (Figure 4E), and deletion of SLC1A3 selectively impeded proliferation in these cells under glutamine starvation but did not affect growth in fully fed conditions (Figure 4F). We also found that deletion of SLC1A3 reduced the growth of MDA-MB-468 and IGROV1 xenograft tumors (Figures 4G and 4H), consistent with glutamine limitation in tumors *in vivo* (Kamphorst et al., 2015).

While the p53 status of IGROV1 cells is unclear, our cells retained wild-type p53 function as measured by the induction of the canonical p53-target genes p21 and MDM2 in response to the p53-activator Nutlin (Figure S4E). Depletion of p53 in this cell line resulted in decreased levels of SLC1A3 protein, consistent with a role for p53 in maintaining SLC1A3 expression (Figure S4F). However, although SLC1A3 levels decreased compared with control, high levels remained and we were unable to detect a growth defect in p53-deleted cells in glutamine-free medium (Figure S4G). OVCAR-4 and MDA-MB-468 both express mutant p53 (L130V and R273H, respectively), suggesting that the increased expression of SLC1A3 in response to glutamine starvation may become uncoupled from wild-type p53, or that some tumor-derived p53 mutants retain the ability to support SLC1A3 expression. Interestingly, depletion of mutant p53 from MDA-MB-468 cells reduced the expression of SLC1A3 (Figure S5C), suggesting that this mutant retains the ability to induce SLC1A3 expression. A previous study showed that several tumor-derived p53 point mutants retain the ability to support cells under glutamine starvation (Tran et al., 2017). Consistently, we found that re-expression of p53 248W (but not another tumor hot-spot mutant p53 175H) in p53-null HCT116 cells rescued growth in the absence of glutamine (Figure S5A). The p53 248W mutant also retained the ability to support expression of both p21 and SLC1A3 (Figure S5B), an activity not exhibited by the 175H mutant, consistent with the failure of this mutant to support growth under glutamine starvation.

### Functions of SLC1A3 under Glutamine Starvation

**Figure 5:**
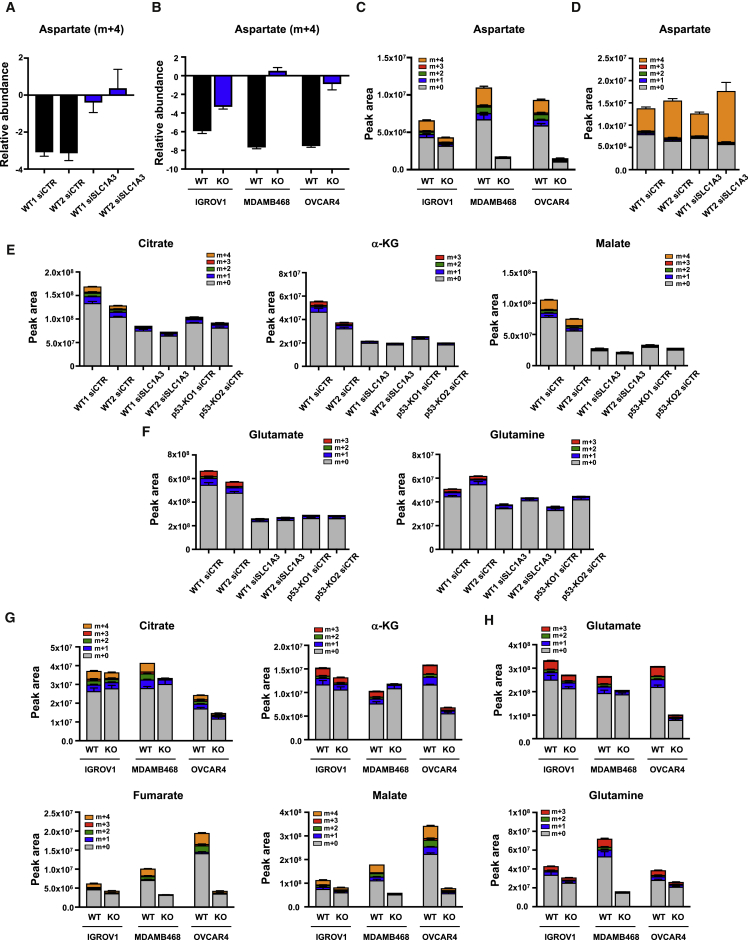
SLC1A3 Depletion Reduces Aspartate Uptake and TCA Activity under Glutamine Deprivation, Phenocopying Loss of p53 (A) HCT116 WT cells transiently depleted of SLC1A3 using siRNA were grown for 2 days in glutamine-free medium and pulsed with [U-^13^C]aspartate for the final 24 hr. Extracellular levels of aspartate (m+4), normalized to cell number, were quantified over 24 hr. Data are presented as mean ± SEM of one representative experiment (averages of triplicate wells). (B) IGROV1, MDA-MB-468, and OVCAR4 WT and SLC1A3 KO cells were grown for 3 days in glutamine-free medium and pulsed with [U-^13^C]aspartate for the final 16 hr. Extracellular levels of aspartate (m+4), normalized to cell number, were quantified over 16 hr. Data are presented as mean ± SEM of one representative experiment (averages of triplicate wells). (C) IGROV1, MDA-MB-468, and OVCAR4 WT and SLC1A3 KO cells were grown for 3 days in glutamine-free medium and pulsed with [U-^13^C]aspartate for the final 16 hr. Intracellular aspartate level was analyzed. Data are presented as mean ± SEM of one representative experiment (averages of triplicate wells). (D–F) HCT116 WT cells transiently depleted of SLC1A3 using siRNA were grown for 2 days in glutamine-free medium and pulsed with [U-^13^C]aspartate for the final 16 hr. Stable isotopomer tracing analysis of intracellular aspartate (D), TCA-cycle intermediates (E), and glutamate and glutamine levels (F) is shown. Data are presented as mean ± SEM of one representative experiment (averages of triplicate wells). (G and H) IGROV1, MDA-MB-468, and OVCAR4 WT and SLC1A3 KO cells were grown for 3 days in glutamine-free medium and pulsed with [U-^13^C]aspartate for the final 16 hr. Stable isotopomer tracing analysis of intracellular TCA-cycle intermediates (G) and glutamate and glutamine levels (H) is shown. Data are presented as mean ± SEM of one representative experiment (averages of triplicate wells). See also Figure S6.

In the CNS, SLC1A3 is expressed at the plasma membrane and functions to remove glutamate from the extracellular space, and ectopic expression of SLC1A3 can promote aspartate uptake (Arriza et al., 1994). In HCT116 cells, knockdown of SLC1A3 decreased the depletion of aspartate from the medium (Figure 5A) without preventing serine or alanine depletion (Figure S6A), similar to the effect of loss of p53 (Figure 3A). SLC1A3 deletion also reduced or prevented the depletion of extracellular aspartate by IGROV1, MDA-MB-468, and OVCAR4 cells under glutamine starvation (Figure 5B), accompanied by a clear drop in intracellular aspartate levels in the SLC1A3-null cells (Figure 5C). These observations are consistent with a function for SLC1A3 in allowing IGROV1, MDA-MB-468, and OVCAR4 cells to take up extracellular aspartate. By contrast, however, intracellular aspartate levels were not substantially lower in SLC1A3-depleted HCT116 cells (Figure 5D), suggesting that these cells retain some ability to import aspartate but fail to utilize it. A similar maintenance of intracellular aspartate was also seen in p53-null HCT116 cells (Figure S6B). Furthermore, removal of aspartate from the medium of wild-type p53 HCT116 cells did not impair growth under glutamine starvation (Figure S6C), suggesting again that the SLC1A3-dependent response in these cells was not a reflection of aspartate import.

Further analysis of these cells showed that loss of SLC1A3 in HCT116, IGROV1, MDA-MB-468, or OVCAR4 cells led to a reduction in overall and aspartate-derived TCA intermediates very similar to that seen in p53-null cells (Figures 5E–5G) and to a reduction in glutamate and glutamine levels (Figures 5F–5H). Furthermore, SLC1A3-depleted, glutamine-starved HCT116 cells show a defect in purine and pyrimidine synthesis (Figure S6D), again very comparable with that seen in p53-null HCT116 cells. The data suggest a defect in aspartate metabolism in all SLC1A3-depleted cells, reflecting an inability to import aspartate in IGROV1, MDA-MB-468, and OVCAR4 cells. However, the maintenance of intracellular aspartate in HCT116 cells suggested that SLC1A3 carries out a different function in these cells.

**Figure 6:**
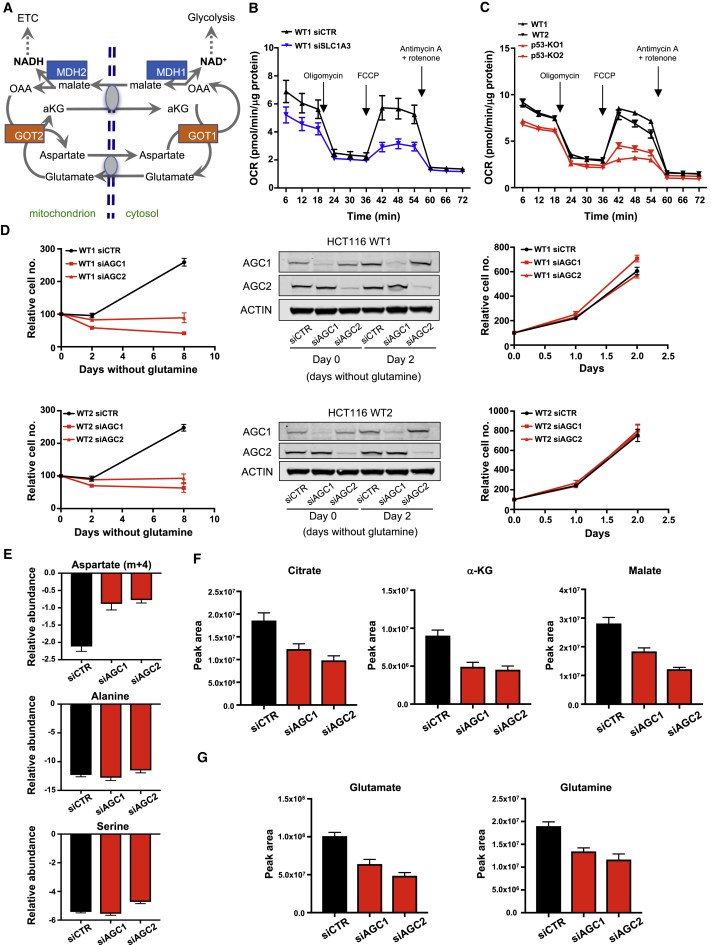
Deletion of SLC1A3 Impedes the ETC and Phenocopies Depletion of the Mitochondrial Aspartate Transporters AGC1 and AGC2 under Glutamine Deprivation (A) Schematic representation of the malate-aspartate shuttle (MAS). In brief, the MAS is a system that allows the transfer of electrons from cytosolic NADH to produce mitochondrial NADH where it is oxidized in the ETC. In the cytoplasm MDH1 catalyzes the reduction of oxaloacetate (OAA), where it accepts an electron from NADH to produce malate and NAD^+^. Malate can then enter the mitochondria where it is oxidized by MDH2 to OAA, resulting in the formation of mitochondrial NADH. Mitochondrial OAA is transaminated into aspartate by GOT2 whereby aspartate exits the mitochondria in exchange for cytosolic glutamate through a carrier. OAA is recovered in the cytosol by GOT1. By coupling aspartate-glutamate exchange, the aspartate-glutamate carrier is essential for the shuttle and is thought to represent the rate-limiting step. (B) Respiratory profiles of HCT116 WT cells transiently depleted of SLC1A3 and grown for 24 hr in glutamine-free medium, in the presence of mitochondrial inhibitors (oligomycin, FCCP [carbonyl cyanide-4-(trifluoromethoxy)phenylhydrazone] antimycin A/rotenone). Arrows indicate incubation of cells with the indicated inhibitors. Data are presented as mean ± SEM of one representative experiment (n = 6 wells). (C) Oxygen consumption rates (OCR) of HCT116 WT and p53-null clones 2 days after glutamine deprivation as in (B). Data are presented as mean ± SEM of one representative experiment (n = 6 wells). (D) Proliferation of HCT116 WT cells transiently depleted of AGC1 or AGC2 using siRNA and cultured in glutamine-free condition (LHS) or complete medium (RHS). Data are presented as mean ± SEM of one representative experiment (averages of triplicate wells). The downregulation of these two proteins was confirmed by western blot (middle panel). (E) HCT116 WT cells transiently depleted of AGC1 or AGC2 using siRNA were grown for 2 days in glutamine-free medium and pulsed with [U-^13^C]aspartate for the final 24 hr. Extracellular levels of aspartate (m+4), alanine, and serine normalized to cell number were quantified over 24 hr. Data are presented as mean ± SEM of one representative experiment (averages of triplicate wells). (F and G) HCT116 WT cells transiently depleted of AGC1 or AGC2 using siRNA were grown for 2 days in glutamine-free medium and pulsed with [U-^13^C]aspartate for the final 24 hr. Intracellular TCA-cycle intermediates (F) and glutamate and glutamine levels (G) were measured. Data are presented as mean ± SEM of one representative experiment (averages of triplicate wells). See also Figure S7.

In considering an alternative role for SLC1A3, we noted previous work that showed SLC1A3 localized to the inner mitochondrial membrane, where it contributes to the malate-aspartate shuttle (MAS) (Ralphe et al., 2004, Ralphe et al., 2005). The MAS normally functions to transfer reducing equivalents between the cytosolic and mitochondrial compartments, thereby supporting both glycolysis and the ETC (Figure 6A). Interestingly, GOT1 and GOT2 are key components of the MAS, and their importance under glutamine starvation (Figure 3E) suggests a role for this shuttle under these conditions.

A critical role for the MAS is to support the ETC. Glutamine-starved, SLC1A3-depleted HCT116 cells showed a drop in oxygen consumption rate (OCR) compared with wild-type cells (Figure 6B), suggesting that SLC1A3 can help to support the ETC and consistent with a role for SLC1A3 in the MAS. A similar reduction in OCR was also seen in glutamine-starved p53-null HCT116 cells (Figure 6C). The ability of p53 to support oxidative phosphorylation has been ascribed to the regulation of SCO2 (Matoba et al., 2006). However, we were unable to detect any change in *SCO2* expression following glutamine withdrawal or p53 deletion (Figure S7A). Fully fed conditions supported a much higher OCR than seen under glutamine depletion that was not affected by loss of p53 (Figure S7B). These results suggest that the MAS becomes important under glutamine-limiting conditions, and can be supported by p53 and SLC1A3. Importantly, a defect in the ETC would also affect the TCA cycle, as seen in both p53- and SLC1A3-depleted cells. Consistently, depletion of SLC1A3 in these cells also resulted in an increase in the NAD^+^/NADH ratio (Figure S7C).

To investigate the role of the MAS more closely, we depleted p53 wild-type HCT116 cells of the two canonical mitochondrial transporters, AGC1 (Aralar; SLC25A12) and AGC2 (Citrin; SLC25A13), which function to exchange mitochondrial aspartate for cytosolic glutamate (Figure 6D). Neither of these transporters showed strong glutamine- or p53-dependent expression in these cells, although AGC2 was slightly induced by glutamine starvation in p53 wild-type but not p53-null cells (Figure S7D). Depletion of either AGC1 or AGC2 had no impact on cells under fully fed conditions but inhibited proliferation of these cells under glutamine starvation (Figure 6D), as seen in the p53- and SLC1A3-depleted cells (Figures 1E and 4D). Interestingly, knockdown of AGC1 or AGC2 also decreased the ability of the cells to deplete aspartate from the medium without affecting alanine or serine uptake under glutamine starvation (Figure 6E). Similarly, AGC1 or AGC2 knockdown also decreased TCA-cycle intermediates (Figure 6F) and glutamate and glutamine levels (Figure 6G). Taken together, our data suggest that SLC1A3 can both promote aspartate uptake and—in some cells—allow aspartate-glutamate exchange at the mitochondria. By supporting aspartate metabolism, SLC1A3 can contribute to nucleotide synthesis and provide reducing equivalents to support the ETC and TCA cycle, so maintaining growth and viability under glutamine starvation.

### Re-expression of SLC1A3 Rescues Viability of p53-Null Cells under Glutamine Starvation

**Figure 7:**
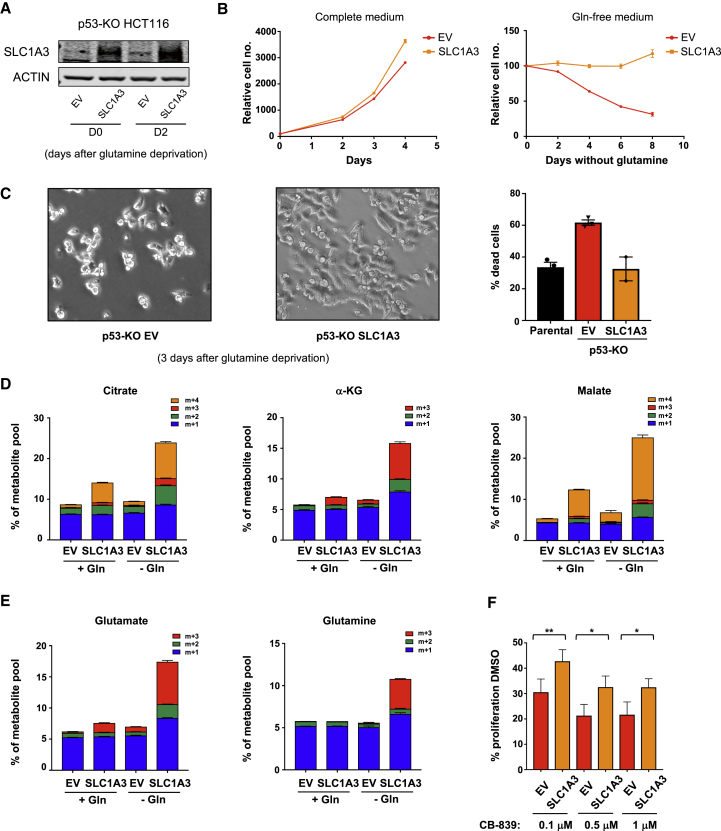
SLC1A3 Re-expression Rescues Survival and Metabolic Deficiency in p53-Null Cells under Glutamine Deprivation (A) p53-null HCT116 cells were infected with a control vector (EV) or a vector encoding SLC1A3 (SLC1A3). Western blot shows efficient SLC1A3 re-expression in these cells when cultured in complete medium (D0) or for 2 days in glutamine-free medium (D2). (B) Proliferation rates of the p53-null cells infected with a control vector or a vector encoding SLC1A3 under complete medium (LHS) or glutamine-free conditions (RHS). Data are presented as mean ± SEM of one representative experiment (averages of triplicate wells). (C) Representative pictures of p53-null cells infected with a control vector or a vector encoding SLC1A3 and cultured for 3 days in glutamine-free medium. Viability of these cells grown for 4 days in glutamine-free medium was assessed by fluorescence-activated cell sorting. Data are presented as mean ± SEM of one representative experiment (averages of triplicate wells). (D) p53-null cells infected with a control vector or a vector encoding SLC1A3 were fed complete medium or glutamine-deficient medium for 2 days in the presence of [U-^13^C]aspartate for the final 16 hr. LC-MS was used for stable isotopomer tracing of TCA-cycle intermediates. Data are presented as mean ± SEM of one representative experiment (averages of triplicate wells). (E) Analysis of glutamate and glutamine levels in cells treated as in (D). Data are presented as mean ± SEM of one representative experiment (averages of triplicate wells). (F) p53-null cells infected with a control vector or a vector encoding SLC1A3 were cultured for 3 days in complete medium in the presence of the glutaminase inhibitor CB-839 (0.1 μM, 0.5 μM, or 1 μM). The graph shows the percentage of growth compared with the untreated condition (DMSO). Data are presented as mean ± SEM from three independent experiments (^∗^p < 0.05, ^∗∗^p < 0.01, paired two-tailed Student’s t test).

To determine whether lack of SLC1A3 expression was the key defect in p53-null cells, we established a line stably expressing transfected SLC1A3 (Figure 7A). Expression of SLC1A3 had no impact on growth in complete medium, but completely rescued the survival defect in p53-null cells following glutamine starvation (Figures 7B and 7C). Re-expression of SLC1A3 in the p53-null cells also promoted the incorporation of aspartate into the TCA-cycle intermediates citrate, α-KG, and malate (Figure 7D) and increased the flux of carbons from aspartate into glutamate and glutamine (Figure 7E), effects that were most pronounced in glutamine-free medium. SLC1A3 reconstituted cells also showed a partial rescue in proliferation in the presence of the glutaminase inhibitor CB-839 (Figure 7F). While other p53 functions may contribute to the adaptation of cells to glutamine starvation, these data highlight the importance of SLC1A3 to this response.

## Discussion

Our identification of SLC1A3 as part of the p53 response contributes to a growing understanding that p53 can support adaptation and survival of cells in response to nutrient depletion. These functions may represent a homeostatic role for p53 beyond cancer development, but the selective retention of protective or adaptive functions of p53 by some cancer-associated p53 mutants is likely to be advantageous during tumor development. Constitutive overexpression of SLC1A3 can also become uncoupled from p53, as has been shown for other p53 target genes that are supportive of cell survival under metabolic stress, such as Tigar (Cheung et al., 2013). Regardless of the mechanism leading to overexpression, tumor cells expressing high levels of SLC1A3 are better able to adapt and survive under low-glutamine conditions.

Aspartate availability has been shown to be important under various conditions of metabolic stress. As an aspartate transporter, SLC1A3 contributes to aspartate metabolism through a number of mechanisms. Most simply, SLC1A3 can function to import extracellular aspartate under conditions of limiting glutamine which, by restricting TCA-cycle and ETC activity, severely impedes *de novo* aspartate synthesis (Birsoy et al., 2015, Sullivan et al., 2015). By importing extracellular aspartate, SLC1A3 can provide cytosolic aspartate, which is critically important for nucleotide synthesis (Lane and Fan, 2015). Indeed, we show that depletion of SLC1A3 results in a defect in both purine and pyrimidine synthesis. However, in HCT116 cells loss of SLC1A3 does not clearly affect intracellular aspartate levels but has profound effects on TCA-cycle and ETC activity, consistent with the previously described role of SLC1A3 as part of the MAS (Ralphe et al., 2004, Ralphe et al., 2005). Supporting this suggestion is the remarkable consistency in phenotype following knockdown of either of the two canonical mitochondrial aspartate-glutamate carriers AGC1, AGC2, or SLC1A3. Interestingly, depletion of each of the transporters results in a loss of ability to grow without glutamine. The basis underlying this lack of redundancy is not clear, and may point to some subtle differences in activity of the three proteins. Our data complement an accompanying study that shows a role for AGC1 in supporting cells under low-glutamine conditions (Alkan et al., 2018).

Our results are consistent with a model in which glutamine starvation results in an increased dependence on aspartate metabolism to support nucleotide synthesis and the TCA cycle—both indirectly by sustaining ETC activity through the MAS and directly as an anaplerotic source—thus allowing for the *de novo* production of glutamate and glutamine (Figure S7E). This model does not exclude other contributions of the MAS to the ability of cells to survive glutamine starvation, such as the provision of cytosolic NAD^+^ to sustain glycolysis.

In KRas mutant pancreas cancers, components of the MAS are used to convert glutamate-derived aspartate into malate, a substrate for NADPH production through malic enzyme. This pathway contributes to antioxidant defense and survival of the KRas transformed cells (Son et al., 2013). However, we were unable to detect a substantial contribution of malic enzyme to the survival of wild-type p53-expressing cells (data not shown), suggesting that NADPH production through this pathway is not the critical activity of SLC1A3.

Previous studies have suggested a role for the MAS in cancer development, with evidence for upregulation of the canonical aspartate-glutamate transporter AGC1 in tumors (Amoedo et al., 2016). We show here that depletion of SLC1A3 can retard tumor growth *in vivo*, raising the possibility that SLC1A3 inhibitors may be useful in cancer therapy. SLC1A3 is predominantly express in astrocytes where it allows glutamate uptake from the neuronal synapse, thereby regulating neuronal excitability (Sery et al., 2015). It is therefore possible that SLC1A3 inhibition would lead to neuronal toxicity. Indeed, a few patients with episodic ataxia have been shown to carry mutations in *SLC1A3* (de Vries et al., 2009), and dysregulation of *SLC1A3* may also be a risk factor for schizophrenia (Walsh et al., 2008). However, SLC1A3 is not the only aspartate/glutamate transporter expressed by glial cells, and other members of the SLC1 family may compensate for SLC1A3 loss (Stoffel et al., 2004). Indeed, glial GLT1-EAAT2 is thought to be the most important glutamate transporter for detoxification of glutamate in the CNS (Tanaka et al., 1997). Transient SLC1A3 expression is also important for stem cell activation in the skin (Reichenbach et al., 2018), although SLC1A3-null mice are generally viable and developed normally, despite showing evidence of neurological abnormalities (Karlsson et al., 2009, Watase et al., 1998). Inhibitors of SLC1A3 that do not cross into the CNS, such as UCPH-101 (Erichsen et al., 2010), may have efficacy in the inhibition of cancer growth without profound general toxicity.

### Limitations of Study

Our cell-culture work was carried out using complete removal of glutamine, a situation unlikely to be encountered *in vivo*. It also seems clear that the ability of p53 to support cells under glutamine depletion will reflect the combined activity of a number of p53 target genes, not only SLC1A3. While we show an effect of SLC1A3 depletion on tumor development in xenograft models, it is possible that this may differ in immunocompetent animals or in humans.

## STAR★Methods

### Key Resources Table

**Table:** 

REAGENT or RESOURCE	SOURCE	IDENTIFIER
**Antibodies**		

Rabbit monoclonal anti-SLC1A3 (D20D5)	Cell Signaling Technology	Cat#5685S; RRID: AB_10694915
Rabbit monoclonal anti-AGC1 (D5I6I)	Cell Signaling Technology	Cat#64169
Rabbit monoclonal anti-MDH2 (D8Q5S)	Cell Signaling Technology	Cat#11908
Rabbit polyclonal anti-Phospho-p53 (Ser15)	Cell Signaling Technology	Cat#9284; RRID: AB_331464
Anti-rabbit IgG	Cell Signaling Technology	Cat#7074; RRID: AB_2099233
Mouse monoclonal anti-p53 (DO-1)	Santa Cruz Biotechnology	Cat#sc-126; RRID: AB_628082
Rabbit polyclonal anti-p21 (C-19)	Santa Cruz Biotechnology	Cat#sc-397; RRID: AB_632126
Goat polyclonal anti-Actin (I-19)	Santa Cruz Biotechnology	Cat#sc-1616
Mouse monoclonal anti-AGC2 (D-7)	Santa Cruz Biotechnology	Cat#sc-393303
Mouse monoclonal anti-MDM2 (SMP14)	Santa Cruz Biotechnology	Cat#sc-965; RRID: AB_627920
Rabbit monoclonal anti-GOT1 (EPR12145)	Abcam	Cat#ab170950
Mouse monoclonal anti-GOT2	Abcam	Cat#ab90562; RRID: AB_2294946
Rabbit monoclonal anti-MDH1 (EPR13597(B))	Abcam	Cat#ab180152
Mouse monoclonal anti-Glutamine Synthetase	BD Biosciences	Cat#610517; RRID: AB_397879

**Chemicals**, **Peptides**, **and Recombinant Proteins**		

CB-839 (for *in vitro* use)	Focus Biomolecules	Cat#10-4556; CAS: 1439399-58-2
CB-839 (for *in vivo* use)	Calithera Bioscience (MTA)	N/A
Poly-D-lysine hydrobromide	Sigma-Aldrich	Cat#P6407; CAS: 27964-99-4
Oligomycin A	Sigma-Aldrich	Cat#75351; CAS: 579-13-5
FCCP	Sigma-Aldrich	Cat#C2920; CAS: 370-86-5
Rotenone	Sigma-Aldrich	Cat# R8875; CAS: 83-79-4
Antimycin A from *Streptomyces* sp.	Sigma-Aldrich	Cat#A8674; CAS: 1397-94-0
D-Glucose (U-13C6, 99%)	Cambridge Isotope Laboratories, Inc.	Cat#CLM-1396; CAS: 110187-42-3
L-Aspartic acid (13C4, 99%)	Cambridge Isotope Laboratories, Inc.	Cat#CLM-1801-H; CAS: 55443-54-4
L-Aspartic acid (15N, 98%)	Cambridge Isotope Laboratories, Inc.	Cat#NLM-718; CAS: 3715-16-0
L-Alanine (13C3, 99%; 15N, 99%)	Cambridge Isotope Laboratories, Inc.	Cat#CNLM-534-H; CAS: 202407-38-3
Dimethyl 2-oxoglutarate	Sigma-Aldrich	Cat#349631; CAS: 13192-04-6
EmbryoMax Nucleosides (100X)	Merck Millipore	Cat#ES-008-D
Nutlin-3	Sigma-Aldrich	Cat#N6287; CAS: 548472-68-0
Fixable Viability Dye eFluor 780	eBioscience	Cat#65-0865-14

**Critical Commercial Assays**		

In-Fusion HD EcoDry Cloning Kit	Clontech	Cat#639689
RNeasy Mini kit	Qiagen	Cat#74104
TruSeq RNA LT Kit v2	Illumina	Cat#RS-122-2001

**Deposited Data**		

Data of the RNA-seq	This paper	GEO: GSE116087

**Experimental Models**: **Cell Lines**		

Human: HCT 116	ATCC	CCL-247
Human: RKO	ATCC	CRL-2577
Human: U2OS	ATCC	HTB-96
Human: A549	ATCC	CCL-185
Human: A375	ATCC	CRL-1619
Human: SiHa	ATCC	HTB-35
Human: RT4	ATCC	HTB-2
Human: MDA-MB-468	The Francis Crick Institute – Cell Services	N/A
Human: OVCAR-4	The Francis Crick Institute – Cell Services	N/A
Human: HEK293T	The Francis Crick Institute – Cell Services	N/A
Human: Phoenix-ECO	The Francis Crick Institute – Cell Services	N/A
Human: IGROV-1	NCI – Tumor Repository	N/A
Human: OVCAR-5	NCI – Tumor Repository	N/A
Human: A2780	European Collection of Authenticated Cell Cultures	Cat#93112519

**Experimental Models**: **Organisms/Strains**		

Mouse: BALB/c Nude	Charles River	Strain Code: 194
Mouse: CD-1 Nude	Charles River	Strain Code: 086
Mouse: Athymic Nude, nu/nu	The Jackson Laboratory	002019

**Oligonucleotides**		

SMARTpool: siGENOME Human SLC1A3 siRNA	Dharmacon	M-007427-00-0020
SMARTpool: siGENOME Human SLC25A12 siRNA	Dharmacon	M-007471-01-0020
SMARTpool: siGENOME Human SLC25A13 siRNA	Dharmacon	M-007472-01-0020
SMARTpool: siGENOME Human GOT1 siRNA	Dharmacon	M-011673-01-0020
SMARTpool: siGENOME Human GOT2 siRNA	Dharmacon	M-011674-02-0020
SMARTpool: siGENOME Human TP53 siRNA	Dharmacon	M-003329-03-0020
siGENOME Non-Targeting siRNA Pool #2	Dharmacon	D-001206-14-20

**Recombinant DNA**		

pX335-U6-Chimeric_BB-CBh-hSpCas9n(D10A)	Cong et al., 2013	Addgene Plasmid #42335
pX335-p53-null-1	This paper	N/A
pX335-p53-null-2	This paper	N/A
lentiCRISPR v2	Sanjana et al., 2014	Addgene Plasmid #52961
lentiCRISPRv2-SLC1A3 -1	This paper	N/A
lentiCRISPRv2-SLC1A3 -2	This paper	N/A
lentiCRISPRv2-SLC1A3 -3	This paper	N/A
pLentiCRISPRv2 crRNA1: AGCACCCACAAGCGTTTCGT	GenScript	N/A
pLentiCRISPRv2 crRNA2: AGCTCATTCTGTATGGTCGG	GenScript	N/A
pLentiCRISPRv2 crRNA3: GACTCTTACCCGAATCACAG	GenScript	N/A
pBABE-hygro	Morgenstern and Land, 1990	Addgene Plasmid #1765
pBABE-hygro-SLC1A3	This paper	N/A
P2A-iRFP IRES puro	Saverio Tardito Lab (Cancer Research UK Beatson Institute)	Tardito et al., 2015
GS-P2A-iRFP IRES puro	Saverio Tardito Lab (Cancer Research UK Beatson Institute)	Tardito et al., 2015
pWZL-ecotropic receptor-neo	N/A	McConnell et al., 1998
pWZL blast p53R175H	Karen Vousden Lab (Francis Crick institute)	Stindt et al., 2015
pWZL blast p53R248W	Karen Vousden Lab (Francis Crick institute)	Stindt et al., 2015
pBABE iRFP IRES puro	Karen Vousden Lab (Francis Crick institute)	Hock et al., 2014
psPAX2	Addgene	Addgene Plasmid #12260
VSV.G	Reya et al., 2003	Addgene Plasmid #14888

**Software and Algorithms**		

GraphPad Prism 7	GraphPad software	N/A
FlowJo software v.10.3	FlowJo	N/A
CRISPR Design Tool	Feng Zhang Lab (MIT)	http://tools.genome-engineering.org
CRISPOR Design Tool	Tefor Infrastructure	http://crispor.tefor.net/crispor.py
IPA	Qiagen	https://www.qiagenbioinformatics.com/products/ingenuity-pathway-analysis/
TopHat2	Kim et al., 2013	https://ccb.jhu.edu/software/tophat/index.shtml
HTSeq v.0.10.0	Simon Anders (EMBL Heidelberg)	https://htseq.readthedocs.io/en/release_0.10.0/
DESeq2	Love et al., 2014	https://bioconductor.org/packages/release/bioc/html/DESeq2.html
Cufflinks	Trapnell et al., 2012	http://cole-trapnell-lab.github.io/cufflinks/cuffdiff/
TraceFinder Version 4.1	Thermo Fisher Scientific	OPTON-30626
Image Studio Lite Version 5.2.5	LI-COR	https://www.licor.com/bio/products/software/image_studio/

### Contact for Reagent and Resource Sharing

Further information and requests for resources and reagents should be directed to and will be fulfilled by the Lead Contact, Karen H. Vousden (karen.vousden@crick.ac.uk).

CB-839 for *in vivo* use was obtained under an MTA with Calithera Bioscience.

### Experimental Model and Subject Details

#### Cell Culture

All the cell lines used in this study are of human origin and were cultured at 37°C in a humidified atmosphere of 5% CO2. HCT116 cells (gender: male) were maintained in culture in McCoy's 5A (Modified) medium (Gibco, 26600023) supplemented with 10% FBS and 1% penicillin-streptomycin; IGROV1 (gender: female), OVCAR-4 (gender: female), OVCAR-5 (gender: female) and RT4 (gender: male) cell lines were cultured in RPMI 1640 medium (Thermo Fisher Scientific, 31870) supplemented with 10% FBS, 2 mM glutamine and 1% penicillin-streptomycin; A2780 (gender: female), A375 (gender: female), SiHa (gender: female), MDA-MB-468 (gender: female), U2OS (gender: female), RKO, A549 (gender: male), HEK293T and Phoenix-ECO cells were cultured in DMEM (Thermo Fisher Scientific, 41966) supplemented with 10% FBS and 1% penicillin-streptomycin.

#### Mice

All animal studies were conducted in compliance with UK Home Office approved project license and in accordance with institutional welfare guidelines. For xenograft experiments, BALB/c female nude mice (obtained from Charles River, 7-8 weeks old), CD-1 female nude mice (obtained from Charles River, 8-9 weeks old) and athymic female nude (*nu/nu*) mice (obtained from The Jackson Laboratory, 7-8 weeks old) were used. Mice were housed 5 per cage in a constant temperature (19-23°C) and humidity (55% ± 10%) animal room, with a 12-hour light/dark cycle (lights on at 7:00 am) and were allowed access to food and water *ad libitum*. Mice were allowed to acclimatize for one week prior to the experiment and were randomly assigned to experimental groups.

### Method Details

#### Glutamine Deprivation

For all glutamine-deprivation experiments, cells were cultured in DMEM without glucose, glutamine, and phenol red (ThermoFisher Scientific, A1443001) and supplemented with 10% dialysed FBS (Hyclone, Thermo Scientific), 1% penicillin-streptomycin, phenol red (11 mg/L), glucose (16 mM), sodium pyruvate (65 μM), L-Proline (0.15 mM), L-Alanine (0.15 mM), L-Aspartic acid (0.15 mM), L-Glutamic acid (0.15 mM) and L-Asparagine (0.34 mM). The complete medium corresponds to the previously described medium supplemented with 2 mM glutamine. During nutrient starvation, the medium was replaced every day.

#### Growth Curves

Cells were seeded in 24-well plates (6.10^4^ cells/well for HCT116 and 3-5.10^4^ cells/well for the other cell lines) in their normal medium. The next day, cells were washed with PBS and moved to glutamine-free medium or the corresponding complete medium described above. Medium was replaced every day. For counting, cells were trypsinized, suspended in PBS-EDTA, and counted with a CASY Model TT Cell Counter (Innovatis, Roche Applied Science). Cells were counted before the medium change to assess the starting cell number and the relative cell number at each time point was calculated.

For the growth curve experiment performed in presence of dimethyl-a-ketoglutarate (DMKG), HCT116 cells were seeded in 6-well plates (5.10^5^ cells/well) and treated with 0.15-0.3mM DMKG (diluted in DMSO), refreshed daily. For the growth curve experiment performed with HCT116 p53-KO clones infected with a vector encoding SLC1A3 or its control vector, cells were seeded in 6-well plates (5.10^5^ cells/well). To assess growth with GLS inhibitor, CB-839, HCT116 were seeded in 24-well plates (3.10^4^ cells/well) and grown in RPMI 1640 medium supplemented with 10% FBS, 2 mM glutamine and 1% penicillin-streptomycin with 0.1, 0.5, or 1 μM CB-839 (Focus biomolecules, diluted in DMSO). To measure growth in glutamine-free medium with or without aspartate (0.15 mM), or the growth of HCT116 p53 null cells stably re-expressing the p53 mutants, cells were seeded in 6-well plates (4.10^5^ cells/well). For the growth curve experiment with nucleoside supplementation, 1X of the following nucleosides mix (100X NUCLEOSIDES for ES CELLS, Millipore, ES-008-D) was diluted in glutamine-free medium and refreshed every two days; HCT116 cells were initially seeded in 6-well plates (4.10^5^ cells/well).

#### siRNA Transfection

The siRNA used to target human *TP53*, *SLC1A3*, *GOT-1*, *GOT-2*, *SLC25A12*, *SLC25A13*, and the non-targeting siRNA control were all purchased from Dharmacon (siGENOME SMART pool siRNA) and transfected using Lullaby siRNA transfection reagent (OZ Biosciences) for 6-8 hr.

#### CRISPR/Cas9 and Selection

Two guide RNAs (p53-null-1: 5-ACCAGCAGCTCCTACACCGG CGG-3 and p53-null-2: 3-GGT CTACTTCGAGGGTCTTACGG-5) targeting either strand of *TP53* at exon 3 were designed *in silico* using the CRISPR Design Tool (http://tools.genome-engineering.org). pX335-U6-Chimeric_BB-CBh-hSpCas9n(D10A) (a gift from Feng Zhang - Addgene plasmid # 42335; Cong et al., 2013), was linearized using BbsI, then the annealed oligonucleotides were ligated into the vector.

HCT116 were plated at 4 × 10^5^ cells per well in a 6-well plate and transfected with either pX335-p53-null-1 or pX335-p53-null-2 using the GeneJuice Transfection Reagent (Merck, Darmstadt, Germany). 24 hr post transfection, cells were trypsinized and 20 μL of cells were plated in a 15 cm plate. p53-CRISPRed clones were selected using 5 μg/mL Nutlin3 (Sigma-Aldrich, Merck, Darmstadt, Germany) and p53 status was determined by immunoblotting for p53 protein expression.

Lentiviral CRISPR for SLC1A3 guide RNAs (1: CAATGGAGAAGAGCCCAAGA TGG, 2: GCACAAAAGCATTCCGAAAC AGG, 3: CACAGTCACCGCTGTCATTG TGG) were identified *in silico* using the CRISPOR design tool (http://crispor.tefor.net/crispor.py). lentiCRISPR v2 (a gift from Feng Zhang (Addgene plasmid # 52961; Sanjana et al., 2014) was linearized using BsmBI and guides were cloned using In-Fusion HD cloning kit from Clontech (Takara Bio USA, Inc). These vectors (lentiCRISPRv2-SLC1A3-1, lentiCRISPRv2-SLC1A3-2 and lentiCRISPRv2-SLC1A3-3) were used to target SLC1A3 in IGROV1 cells. The three lentiviral plasmids used to target SLC1A3 in MDA-MB-468 and OVCAR4 cells were directly purchased from GenScript and correspond to pLentiCRISPRv2 vector containing the following guide RNAs: AGCACCCACAAGCGTTTCGT (crRNA1), AGCTCATTCTGTATGGTCGG (crRNA2), GACTCTTACCCGAATCACAG (crRNA3).

Lentiviral plasmids together with packaging and envelope plasmids (psPAX2 and VSV.G; Reya et al., 2003) were transfected into HEK293T cells using jetPRIME (Polyplus transfection). After 24 hr incubation with the transfection mix, medium was changed and 48 hr after medium change, the viral particle containing-medium was filtered (0.45 mm pore filter) and mixed with 4 μg/mL Polybrene (Sigma-Aldrich). The medium containing lentiviruses was then incubated with the recipient cells for 24 hr. After lentivirus infection, IGROV1, MDA-MB-468 and OVCAR4 cells were selected with 1.5 μg/mL, 2 μg/mL, and 0.5 μg/mL of Puromycin (Sigma-Aldrich) respectively for 2-3 weeks and analyzed for loss of SLC1A3 by western blot.

#### Stable Re-expression of SLC1A3 and GS

Human *SLC1A3* was cloned into the pBABE-hygro vector (gift from Feng Zhang (Addgene plasmid # 1765; Morgenstern and Land, 1990): pBABE-hygro vector was digested with BamHI and SalI and cDNA coding for human *SLC1A3* (purchased from IDT) cloned using In-Fusion HD cloning kit from Clontech (Takara Bio USA, Inc). The cloned ORF contains silent mutations to generate an siRNA resistant mRNA. pBABE-hygro-*SLC1A3* was sequenced to confirm that no mutations arose during the cloning. Retroviral transduction was performed as described previously (Morgenstern and Land, 1990). In brief, Phoenix-ECO cells were transfected with pBABE-hygro-SLC1A3 using jetPRIME (Polyplus transfection), medium changed after 24 hr. After further 24 hr the supernatant was used to transduce target cells in the presence of polybrene (4 μg/mL). Cells were selected with 0.1 mg/mL Hygromycin B (Thermo Fisher Scientific) for 2-3 weeks and analyzed for re-expression of SLC1A3 by western blot.

Stable transfection of vector containing P2A-iRFP IRES puro only or GS-P2A-iRFP IRES puro in HCT116 cells was performed as described previously (Tardito et al., 2015). HCT116 cells were selected in medium containing 0.5 μg/mL puromycin (Sigma-Aldrich) for 2-3 weeks and analyzed for re-expression of GS by western blot.

#### Generation of Cells Expressing p53 Mutants

HCT116 p53 null clone (p53-KO1) was transiently transfected with a pWZL-ecotropic receptor-neo plasmid (McConnell et al., 1998) using GeneJuice. The next day, these cells were infected with pBABE-ecotropic receptor-neo retroviral particles and selected with G418 for an ecoR positive pool. These cells were then infected with the p53 mutant constructs (pWZL blast p53R175H and pWZL blast p53R248W) described previously (Stindt et al., 2015) and selected with blasticidin (5 μg/mL). Finally, each pool of these cells was infected with retroviral pBABE iRFP IRES puro (Hock et al., 2014) and finally selected with puromycin (0.2 μg/mL).

#### Measuring Cell Death and Viability

HCT116 cells (4× 10^5^) were seeded in triplicate wells of 6-well plates in McCoy’s medium. After 16–24  hr cells were washed with PBS and received glutamine-free medium for the indicated times. Medium was changed every day until 48 hr before cell death analysis. Supernatant and attached cells were analyzed for cell death using the Fixable Viability Dye eFluor 780 (eBioscience) at a 1:1000 dilution. Briefly, cells were trypsinized, pooled with corresponding supernatant and centrifuged at 1300rpm for 5 min. Samples were then stained for 10 min in the dark followed by another spin and re-suspension in PBS. Cells were analyzed using the BD FACSymphony (BD Biosciences). Data were analyzed using FlowJo Software (FlowJo, LLC).

#### RNA-seq Experiment

HCT116 cells were grown for 48 hr in complete medium or in glutamine-free medium and RNA was extracted using the Qiagen RNeasy Mini kit according to manufacturer's instructions. Quality of the purified RNA was tested on an Agilent 2200 Tapestation using RNA screentape. Libraries for cluster generation and DNA sequencing were prepared following an adapted method from Fisher et al. (2011) using Illumina TruSeq RNA LT Kit v2. Quality and quantity of the DNA libraries was assessed on a Agilent 2200 Tapestation (D1000 screentape) and Qubit (Thermo Fisher Scientific) respectively. The libraries were run on the Illumina Next Seq 500 using the High Output v2. 75 cycle kit (2x36cycles, paired end reads, single index).

Paired-end reads were aligned to the human genome (hg19) using a splicing-aware aligner (TopHat2) (Kim et al., 2013). Only unique reads were retained. Reference splice junctions were provided by a reference transcriptome (Ensembl build 73), and novel splicing junctions determined by detecting reads that spanned exons that were not in the reference annotation. True read abundance at each transcript isoform was assessed using HTSeq (Python) before determining differential expression with the tool DESeq2 (Love et al., 2014), which models mean-variance dependence within the sample set. Significance was determined using an FDR corrected p value <= 0.05. Heatmaps were created using the R statistical package with ‘ggplot2’ and ‘fastcluster’. Gene ontologies were produced using Ingenuity IPA suite. Explicit transcript expression values are provided in the form of FPKM (frequency per kilobase per million mapped reads) using Cufflinks (Trapnell et al., 2012).

#### Liquid Chromatography–Mass Spectrometry

HCT116 cells (0.5 × 10^6^), MDA-MB-468 cells (1.8 × 10^5^), IGROV1 cells (1.8× 10^5^) and OVCAR4 cells (1.8 × 10^5^) were seeded in triplicate wells of 6-well plates in their normal medium. Duplicate plates were seeded for cell counts which were used for normalization of LC-MS analysis. After 16 hr, cells were washed with PBS and moved to complete or glutamine-free medium for the indicated times. For glucose, aspartate, and alanine flux experiments, medium was replaced with complete medium or glutamine-free medium, with glucose substituted for 10 mM U-[^13^C]-glucose (Cambridge Isotopes), with aspartate substituted for 0.15 mM [U-^13^C]L-Aspartic acid (Cambridge Isotopes) or 0.15 mM [^15^N]L-Aspartic acid (Cambridge Isotopes) or with alanine substituted for 0.15 mM [U-^13^C, ^15^N]L-Alanine (Cambridge Isotopes) respectively. LC-MS of intracellular and extracellular metabolites was performed as previously described (Labuschagne et al., 2014). Briefly, cells were washed with PBS before metabolite extraction using ice-cold extraction buffer consisting of methanol, acetonitrile, and H_2_O (50:30:20). For LC-MS analysis on tumor samples, tissue was homogenized at 40 mg tissue/mL of the same extraction buffer at 0°C with the Precellys 24 homogenizer (Bertin Instruments). Homogenized samples were centrifuged (16,000g/10 min/2°C) and the supernatant collected to be centrifuged again (16,000g/10 min/2°C). Supernatant were then collected for analysis.

Metabolites were analyzed by LC-MS using a Dionex Ultimate 3000 LC system coupled to a Q Exactive mass spectrometer (Thermo Scientific). Analytes were separated on a Sequant ZIC-pHILIC column (2.1 × 150 mm, 5 μm) (Merck) using the following elution buffers. Buffer A consisting of Acetonitrile (ACN) and buffer B consisting of 20 mM (NH_4_)_2_CO_3_, 0.1% NH_4_OH in H_2_O. A program with a linear gradient starting at 80% (A) and decreasing to 20% (A) over 17 min was used followed by washing and re-equilibration steps with a total run time of 23.5 min. Ionization occurred in the HESI probe connected to the Q-Exactive which operated in full scan mode over a mass range of 75–1,000 m/z with polarity switching at a resolution of 35,000. Metabolites were analyzed using Thermo TraceFinder software.

Extracellular metabolites were extracted by adding 10 μL of cell culture media to 490 μL of ice cold extraction buffer as mentioned above and vortexed for 20 seconds. Samples were centrifuged at 16,000 x g and supernatants collected for analysis. Extracellular metabolites were measured using an Orbitrap Exactive in line with an Accela autosampler and an Accela 600 pump (Thermo Scientific). Analytes were separated on the same column and elution program as mentioned above. The Exactive operated in full-scan mode with polar switching. Metabolites were analyzed using Thermo TraceFinder software.

#### Metabolic Assays

Oxygen consumption rate (OCR) was measured using an XF96 Extracellular Flux Analyzer (Seahorse Bioscience, North Billerica, MA, USA). Cells were plated at a concentration of 15,000-25,000 cells/well onto XF96 Seahorse plates coated with Poly-D-lysine (50 μg/mL). Cells were grown overnight in complete medium, then medium was replaced with either complete medium or glutamine-free medium for the indicated time. Immediately before assaying, medium was replaced with the XF Base medium Minimal DMEM (Agilent Seahorse XF) supplemented with 1% dialysed FBS, 16 mM glucose, 65 μM sodium pyruvate ± 2 mM glutamine (pH 7.4) and cells were incubated at 37°C in a CO_2_-free incubator for 30-45 min. 1 μM Oligomycin A, 0.5 μM FCCP and 1 μM Rotenon/Antimycin A were added to measure ATP-coupled, maximal, and mitochondrial-dependent basal OCR, respectively. These drugs were purchased from Sigma Aldrich (Merck, Darmstadt, Germany). OCR was normalized to the protein content using the Lowry assay.

#### Western Blot

Protein lysates were prepared in RIPA-buffer (Millipore) supplemented with complete protease inhibitors (Roche) and phosphatase inhibitor cocktail (Thermo Fisher Scientific). Lysates were separated using precast NuPAGE 4-12% Bis-Tris Protein gels (Invitrogen, Life Technologies) and transferred to nitrocellulose membranes. Proteins were then detected with a LI-COR Odyssey Infrared scanner and quantified using Image Studio Lite Version 5.2.5 (LI-COR). For SLC1A3 detection, proteins were detected using SuperSignal West Femto Maximum Sensitivity Substrate (Thermo Fisher Scientific). Primary antibodies used were as follows: SLC1A3 (5685), AGC1 (64169), MDH2 (11908), phospho-p53 (S15) (9284) from Cell Signaling Technology; p53 (DO-1, sc-126), p21 (sc-397), ACTIN (sc-1616), AGC2 (sc-393303), MDM2 (SMP14, sc-965) from Santa Cruz Biotechnology; GOT1 (ab170950), GOT2 (ab90562), MDH1 (ab180152) from Abcam; glutamine synthetase (610517) from BD Transduction Laboratories. Secondary antibodies for the relevant species were IRDye800CW- or IRDye680LT-conjugated (LiCor Biosciences), and for SLC1A3 detection, an Anti-rabbit IgG, HRP-linked antibody was used (Cell Signaling Technology (7074)). ACTIN expression was used as a loading control.

#### *In Vivo* Experiments

BALB/c female nude mice (obtained from Charles River, 7-8 weeks old) received unilateral subcutaneous injections of 200 μL of MDA-MB-468 cells (8 × 10^6^ cells) suspended in phosphate buffered saline (PBS). For IGROV1 xenograft experiment, CD-1 female nude mice (obtained from Charles River, 8-9 weeks old) received unilateral subcutaneous injections of 100 μL of cells (2 × 10^6^ cells) suspended in PBS. Once the tumors were palpable, subcutaneous growth was measured twice a week by caliper. Tumor volume was estimated using the following formula: length^∗^width^2^/2.

For HCT116 xenograft experiments, athymic female nude (*nu/nu*) mice (obtained from The Jackson Laboratory, 7-8 weeks old) received unilateral subcutaneous injections of 100 μL of HCT116 cells (2 × 10^6^ cells) suspended in phosphate buffered saline (PBS). Once the different experimental groups reached an average tumor volume of approximatively 100 mm^3^, mice were treated either with vehicle (25% (w/v) hydroxypropyl- b-cyclodextrin in 10 mmol/L citrate, pH 2) or 200 mg/kg CB-839 (obtained from Calithera Bioscience) prepared in vehicle twice daily by oral gavage for 14 days. Subcutaneous growth was measured twice a week by caliper and tumor volume was calculated using the following formula: length^∗^width^2^/2. 4 hr after the last oral gavage, tumors were harvested and snap frozen for further analysis.

### Quantification and Statistical Analysis

Statistical details of experiments can be found in the figure legends. All data are expressed as mean ± SEM. For *in vitro* experiments, statistical significance was determined using paired two-tailed Student’s t test or Mann-Whitney nonparametric test. For xenograft experiments, statistical significance was determined using two-way ANOVA plus Bonferroni post hoc test. All these statistical analyses were carried out in GraphPad Prism 7. RNA-seq data were analyzed through the use of IPA (Qiagen). p values below 0.05 were considered statistically significant. Significance in all figures is indicated as follows: ^∗^ p < 0.05, ^∗∗^ p < 0.01, ^∗∗∗^ p < 0.001, ^∗∗∗∗^ p < 0.0001, ns: no significance.

### Data and Software Availability

The RNA sequencing row data have been deposited in Gene Expression Omnibus under GEO: GSE116087.

## Supplemental Information

Document S1. Figures S1–S7

Document S2. Article plus Supplemental Information
