# Supplementary material for: A Role for p53 in the Adaptation to Glutamine Starvation through the Expression of SLC1A3
Source: Cell Metab. 2018 Nov 6;28(5):721–736.e6. doi: 10.1016/j.cmet.2018.07.005 (PMC6224545; doi:10.1016/j.cmet.2018.07.005)
Supplement: Document S1. Figures S1–S7 [file mmc1.pdf]

Cell Metabolism, Volume *28*

Supplemental Information

A Role for p53 in the Adaptation to Glutamine Starvation through the Expression of SLC1A3

Mylène Tajan, Andreas K. Hock, Julianna Blagih, Neil A. Robertson, Christiaan F. Labuschagne, Flore Kruiswijk, Timothy J. Humpton, Peter D. Adams, and Karen H. Vousden

Figure S1

A

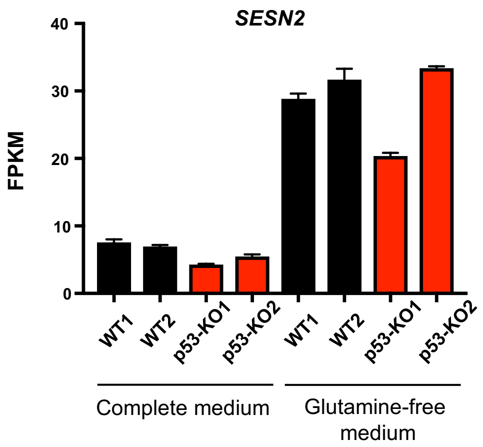

B

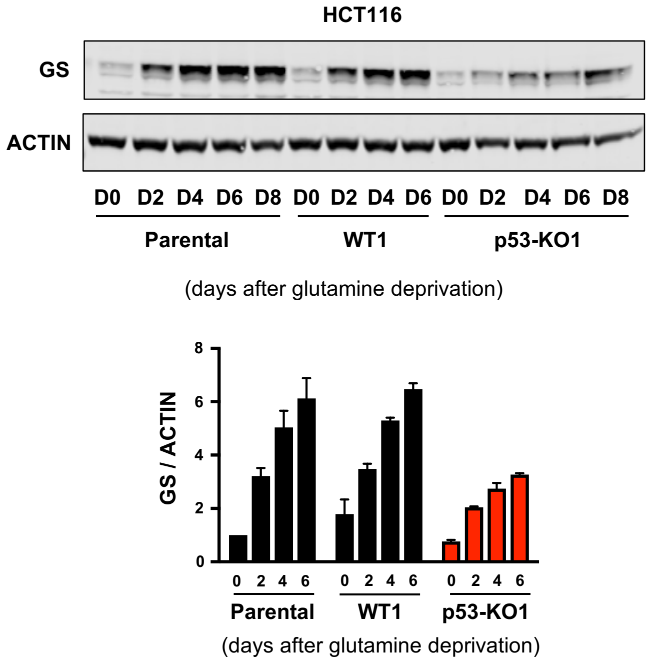

C

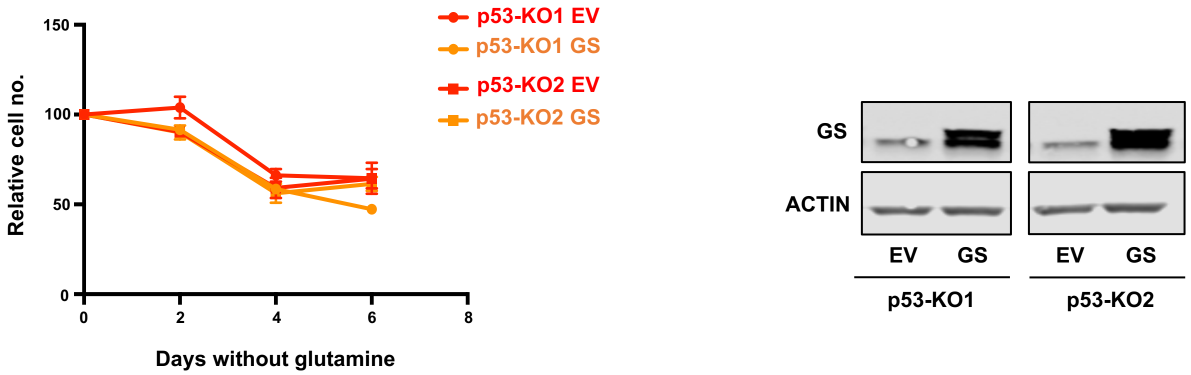

D

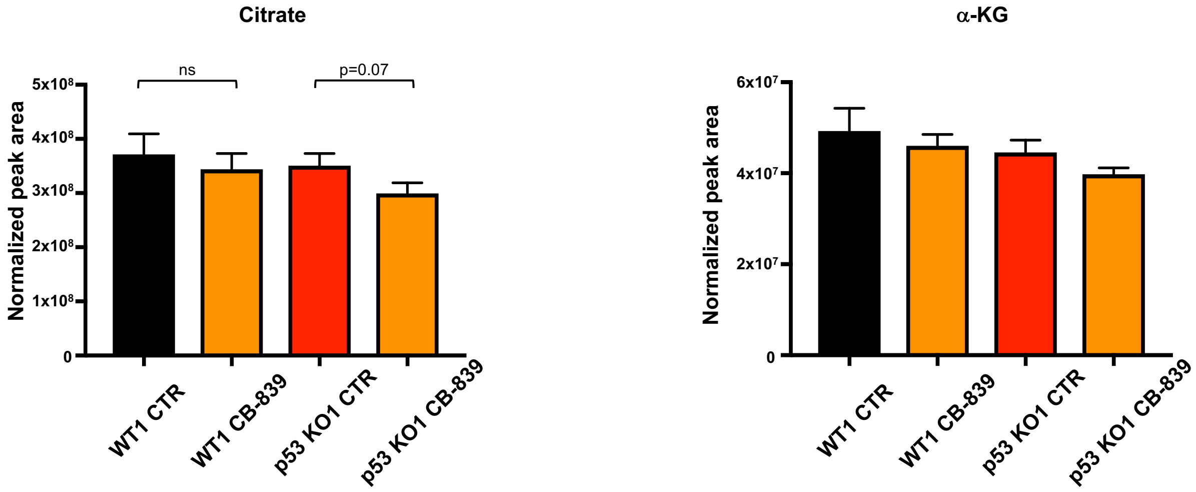

Figure S1 (related to Figure 2): Response of p53 wild type and null cells to glutamine starvation or glutaminase inhibition.

A. Transcriptional expression of *SESN2* in HCT116 WT and p53-null clones grown for 2 days in complete medium or in glutamine-free medium. Data are presented as mean ± SEM (averages of triplicate wells).

B. Western blot analysis demonstrating glutamine synthetase (GS) expression in parental, WT or p53-null clone cultured for 0, 2, 4, 6 or 8 days in glutamine-free condition. Westerns blots from three independent experiments were quantified (bottom). Data are presented as mean ± SEM.

C. p53-null cells infected with a control vector or a vector encoding for GS were grown in glutamine-free medium for 6 days and counted every 2 days (left panel). Data are presented as mean ± SEM of one representative experiment (averages of triplicate wells). Western blot shows stable overexpression of GS in p53-null cells (right panel).

D. Metabolites were extracted from the tumours derived from HCT116 WT1 or p53 KO1 xenografts 4 hours after the final gavage of the nude mice with vehicle (CTR) (n = 7 for WT1; n = 7 for p53 KO1) or CB-839 (n = 8 for WT1; n = 8 for p53 KO1) and normalized to the tumor extract mass. Data are presented as mean ± SEM. (Mann-Whitney nonparametric test).

Figure S2

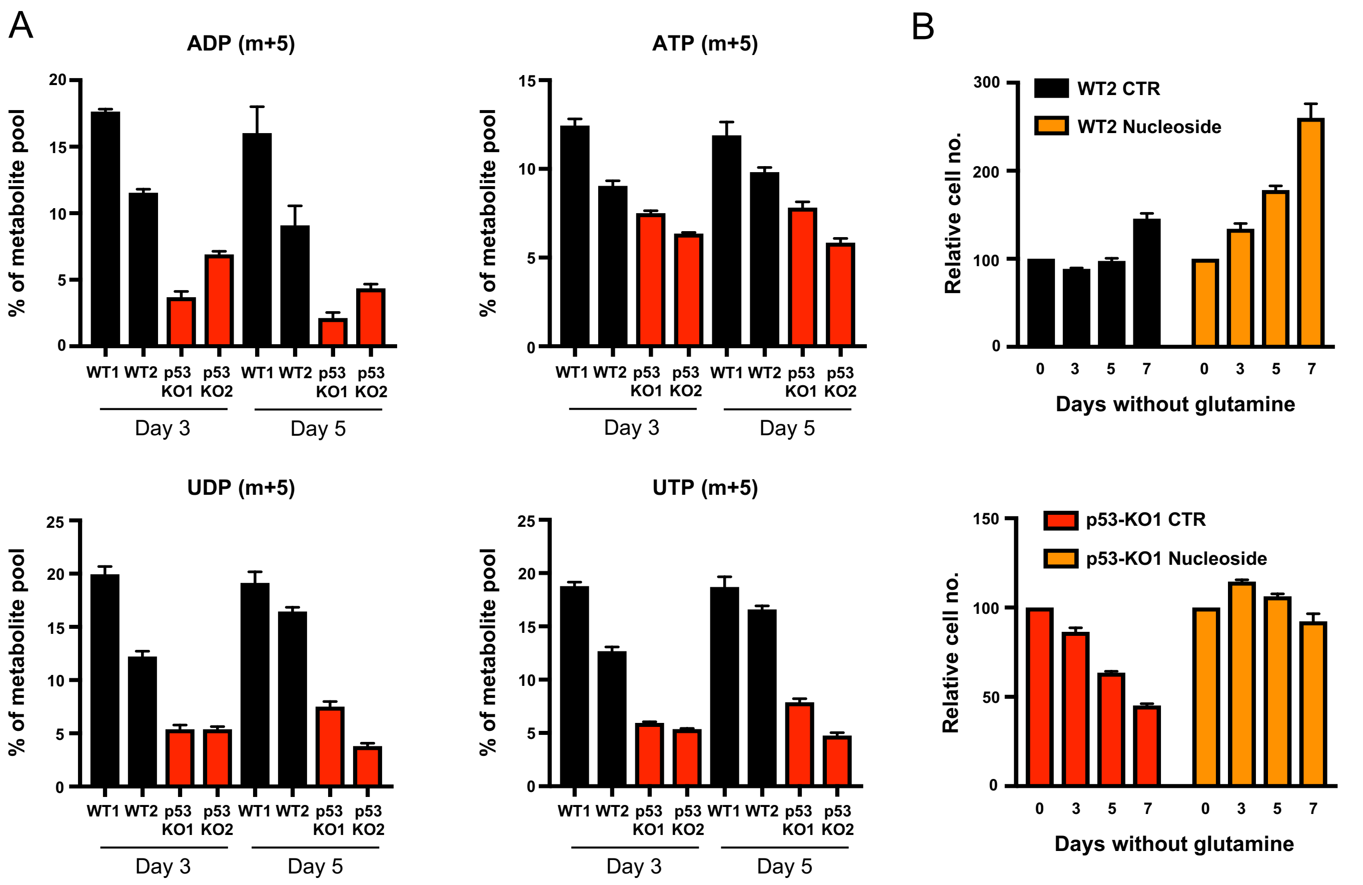

A
ADP (m+5)
ATP (m+5)
UDP (m+5)
UTP (m+5)
% of metabolite pool
WT1 WT2 p53 KO1 p53 KO2 WT1 WT2 p53 KO1 p53 KO2
Day 3
Day 5
B
WT2 CTR
WT2 Nucleoside
p53-KO1 CTR
p53-KO1 Nucleoside
Relative cell no.
Days without glutamine

Figure S2 (related to Figure 3): Nucleotide synthesis is impaired in p53-null cells under glutamine deprivation.

A. HCT116 isogenic cell clones were cultured in glutamine-deficient medium for 3 or 5 days and stable isotopomer tracing analysis with U-[^{13}C]-glucose was performed. Metabolites were extracted and analyzed by LC-MS. Data presented as mean ± SEM of triplicate wells.

B. HCT116 isogenic cell clones grown in glutamine-deficient medium for 7 days were supplemented with or without a nucleoside mix. Total cell numbers were counted regularly. Data are presented as mean ± SEM of one representative experiment (averages of triplicate wells).

Figure S3

A

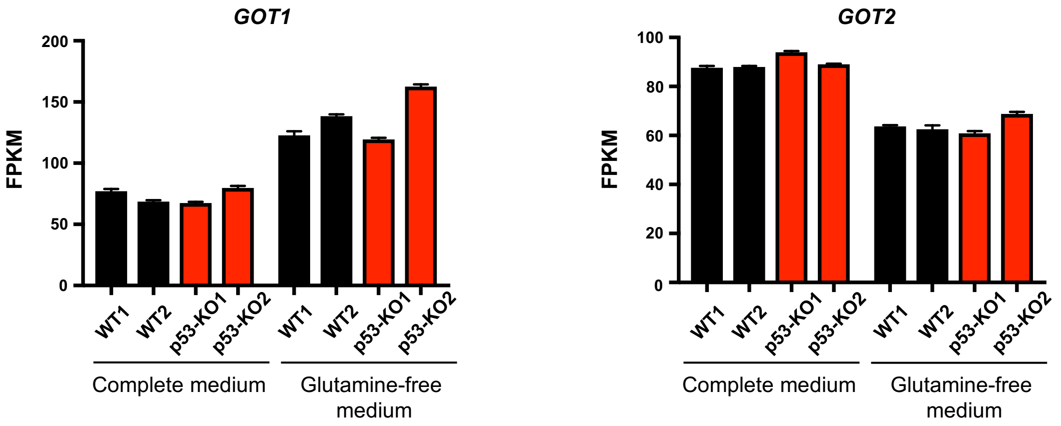
GOT1
GOT2
FPKM
200
150
100
50
0
100
80
60
40
20
WT1
WT2
p53-KO1
p53-KO2
Complete medium
Glutamine-free medium

B

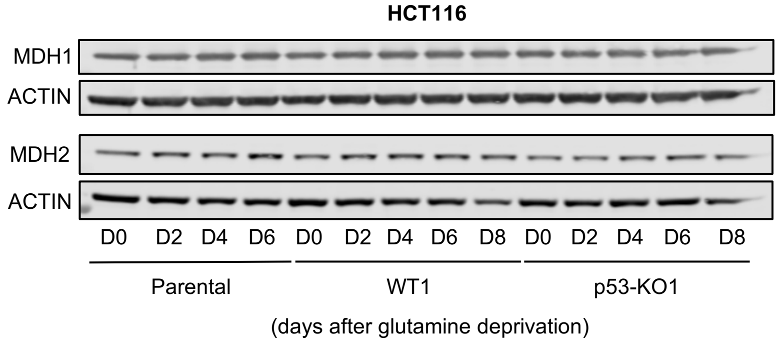
HCT116
MDH1
ACTIN
MDH2
ACTIN
D0
D2
D4
D6
D0
D2
D4
D6
D8
D0
D2
D4
D6
D8
Parental
WT1
p53-KO1
(days after glutamine deprivation)

Figure S3 (related to Figure 3): Expression of *GOT1/2* and MDH1/2 in p53 wild type and null cells under glutamine deprivation.

A. Transcriptional expression of *GOT1* (left) and *GOT2* (right) in HCT116 WT and p53-null clones grown for 2 days in complete medium or in glutamine-free medium. Data are presented as mean ± SEM (averages of triplicate wells).

B. Western blots show MDH1 and MDH2 expression in HCT116 parental, WT and p53-null clones cultured for 0, 2, 4, 6 or 8 days in glutamine-free condition.

Figure S4

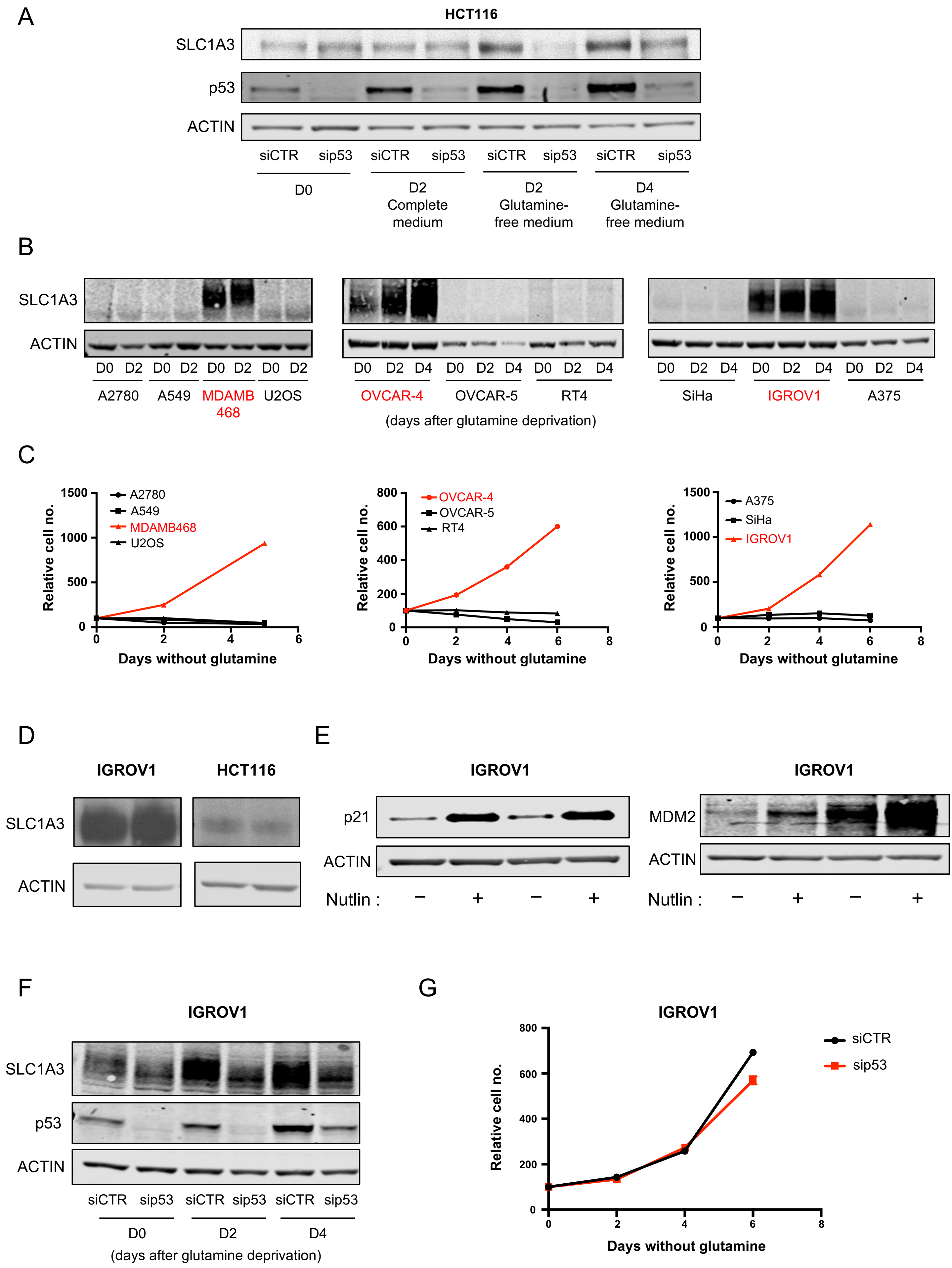
A
HCT116
SLC1A3
p53
ACTIN
siCTR sip53 siCTR sip53 siCTR sip53 siCTR sip53
D0
D2 Complete medium
D2 Glutamine-free medium
D4 Glutamine-free medium
B
SLC1A3
ACTIN
D0 D2 D0 D2 D0 D2 D0 D2
A2780 A549 MDAMB 468 U2OS
D0 D2 D4 D0 D2 D4 D0 D2 D4
OVCAR-4 OVCAR-5 RT4
(days after glutamine deprivation)
D0 D2 D4 D0 D2 D4 D0 D2 D4
SiHa IGROV1 A375
C
Relative cell no.
A2780
A549
MDAMB468
U2OS
OVCAR-4
OVCAR-5
RT4
A375
SiHa
IGROV1
Days without glutamine
D
IGROV1
HCT116
SLC1A3
ACTIN
E
IGROV1
p21
ACTIN
Nutlin : − + − +
IGROV1
MDM2
ACTIN
Nutlin : − + − +
F
IGROV1
SLC1A3
p53
ACTIN
siCTR sip53 siCTR sip53 siCTR sip53
D0 D2 D4
(days after glutamine deprivation)
G
IGROV1
siCTR
sip53
Relative cell no.
Days without glutamine

Figure S4 (related to Figure 4): SLC1A3 expression in human cancer cell lines.

A. p53 was transiently knocked down in HCT116 parental cells using siRNA and cultured for 2 days in complete medium or for 2 and 4 days in glutamine-free medium. Western blots show SLC1A3 and p53 expression over time.

B. Western blots show the expression of SLC1A3 in cell lines derived from diverse cancer types and grown for 0, 2 or 4 days in glutamine-free medium.

C. Proliferation rates of tumor cell lines cultured for 6 days in glutamine-free medium. Data are presented as mean ± SEM (averages of triplicate wells).

D. Western blots showing SLC1A3 expression in IGROV1 and HCT116 cells grown in complete medium.

E. Western blots showing expression of p21 and MDM2, both products of p53-responsive genes, in IGROV1 cells treated with Nutlin (10 µM) for 24 hours.

F. p53 was transiently depleted from IGROV1 cells using siRNA and cultured for 0, 2 or 4 days in glutamine-free medium. Western blots show SLC1A3 and p53 expression over time.

G. Proliferation of IGROV1 cells transiently depleted of p53 using siRNA and cultured for 6 days in glutamine-free medium. Data are presented as mean ± SEM of one representative experiment (averages of triplicate wells).

Figure S5

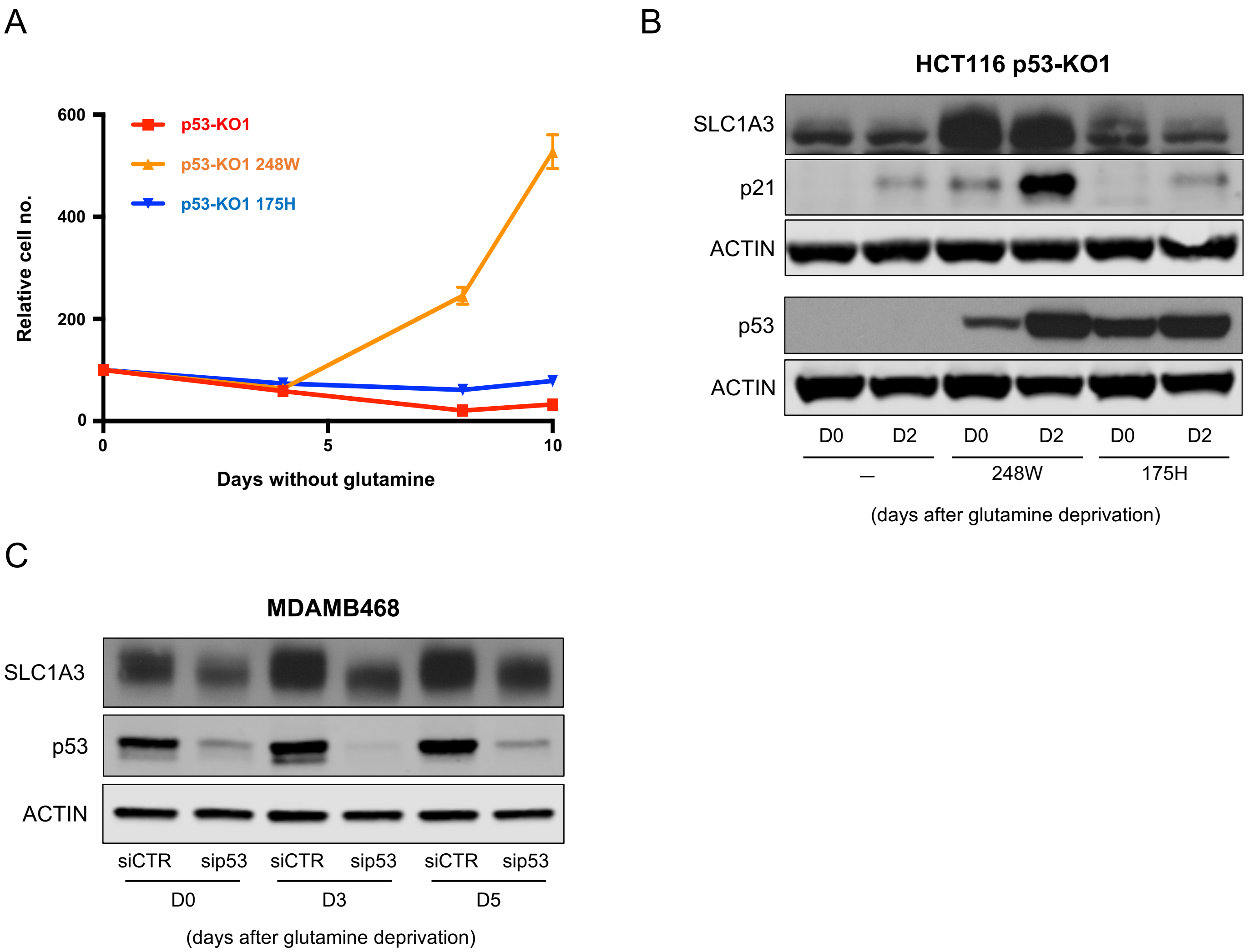
A
600
400
200
0
0
5
10
Relative cell no.
Days without glutamine
p53-KO1
p53-KO1 248W
p53-KO1 175H
B
HCT116 p53-KO1
SLC1A3
p21
ACTIN
p53
ACTIN
D0
D2
D0
D2
D0
D2
—
248W
175H
(days after glutamine deprivation)
C
MDAMB468
SLC1A3
p53
ACTIN
siCTR
sip53
siCTR
sip53
siCTR
sip53
D0
D3
D5
(days after glutamine deprivation)

Figure S5 (related to Figure 4): Rescue of growth under glutamine starvation and SLC1A3 expression by tumor derived p53 mutants.

A. p53 mutants 248W and 175H were stably expressed in p53-KO1 HCT116 cells and these cell lines were grow in glutamine-free condition for 10 days. Data are presented as mean ± SEM of one representative experiment (averages of triplicate wells).

B. Representative western blots showing expression of SLC1A3, p21 and p53 in p53-KO1 HCT116 cells expressing or not p53 mutants, cultured for 0 or 2 days in glutamine-free condition.

C. p53 was transiently depleted from MDA-MB-468 cells using siRNA and cultured for 0, 3 or 5 days in glutamine-free medium. Western blots show SLC1A3 and p53 expression over time.

Figure S6

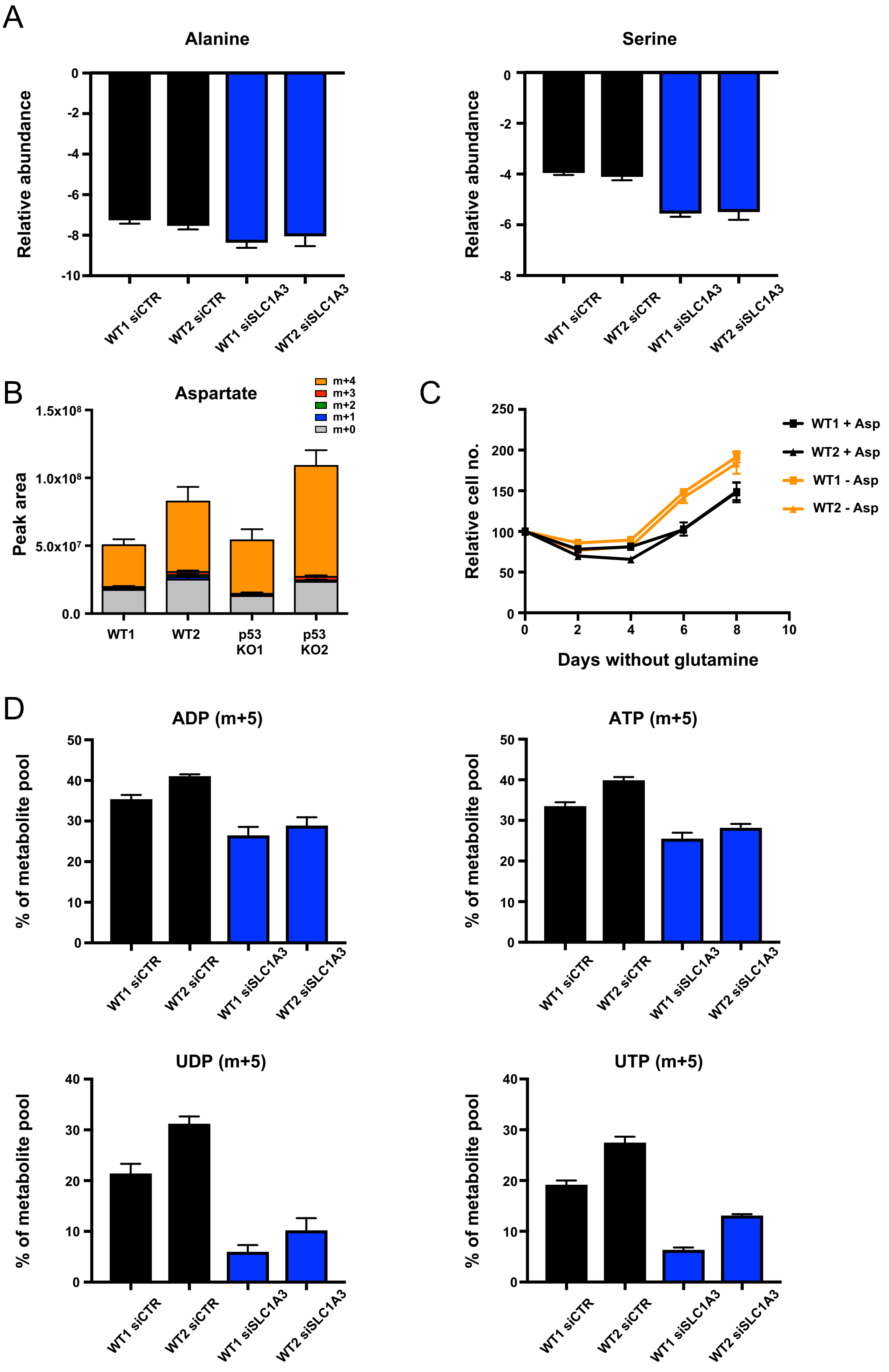

A
Alanine
Relative abundance
0
-2
-4
-6
-8
-10
WT1 siCTR
WT2 siCTR
WT1 siSLC1A3
WT2 siSLC1A3
Serine
Relative abundance
0
-2
-4
-6
-8
WT1 siCTR
WT2 siCTR
WT1 siSLC1A3
WT2 siSLC1A3
B
Aspartate
m+4
m+3
m+2
m+1
m+0
Peak area
1.5x10⁸
1.0x10⁸
5.0x10⁷
0.0
WT1
WT2
p53 KO1
p53 KO2
C
Relative cell no.
250
200
150
100
50
0
0
2
4
6
8
10
Days without glutamine
WT1 + Asp
WT2 + Asp
WT1 - Asp
WT2 - Asp
D
ADP (m+5)
% of metabolite pool
50
40
30
20
10
0
WT1 siCTR
WT2 siCTR
WT1 siSLC1A3
WT2 siSLC1A3
ATP (m+5)
% of metabolite pool
50
40
30
20
10
0
WT1 siCTR
WT2 siCTR
WT1 siSLC1A3
WT2 siSLC1A3
UDP (m+5)
% of metabolite pool
40
30
20
10
0
WT1 siCTR
WT2 siCTR
WT1 siSLC1A3
WT2 siSLC1A3
UTP (m+5)
% of metabolite pool
40
30
20
10
0
WT1 siCTR
WT2 siCTR
WT1 siSLC1A3
WT2 siSLC1A3

Figure S6 (related to Figure 5): The effect of SLC1A3 downregulation on amino acid uptake and nucleotide synthesis under glutamine starvation.

A. HCT116 WT cells transiently depleted of SLC1A3 using siRNA were grown for 2 days in glutamine-free medium and pulsed with U-[^{13}C]-aspartate for the final 24 hours. Extracellular levels of alanine and serine normalized to cell number, were quantified over 24 hours and analyzed by LC-MS. Data are presented as mean ± SEM of one representative experiment (averages of triplicate wells).

B. Stable isotopomer tracing analysis of U-[^{13}C]-aspartate incorporation into cells in HCT116 WT and p53-null cells under glutamine starvation. Data are presented as mean ± SEM of one representative experiment (averages of triplicate wells).

C. HCT116 WT cells were grown for 8 days in glutamine-free medium with or without aspartate (0.15 mM). Data are presented as mean ± SEM of one representative experiment (averages of triplicate wells).

D. HCT116 WT cells transiently depleted of SLC1A3 using siRNA were grown for 2 days in glutamine-free medium and pulsed with U-[^{13}C]-glucose for the final 16 hours. Metabolites were extracted and analyzed by LC-MS. Data are presented as mean ± SEM of one representative experiment (averages of triplicate wells).

Figure S7

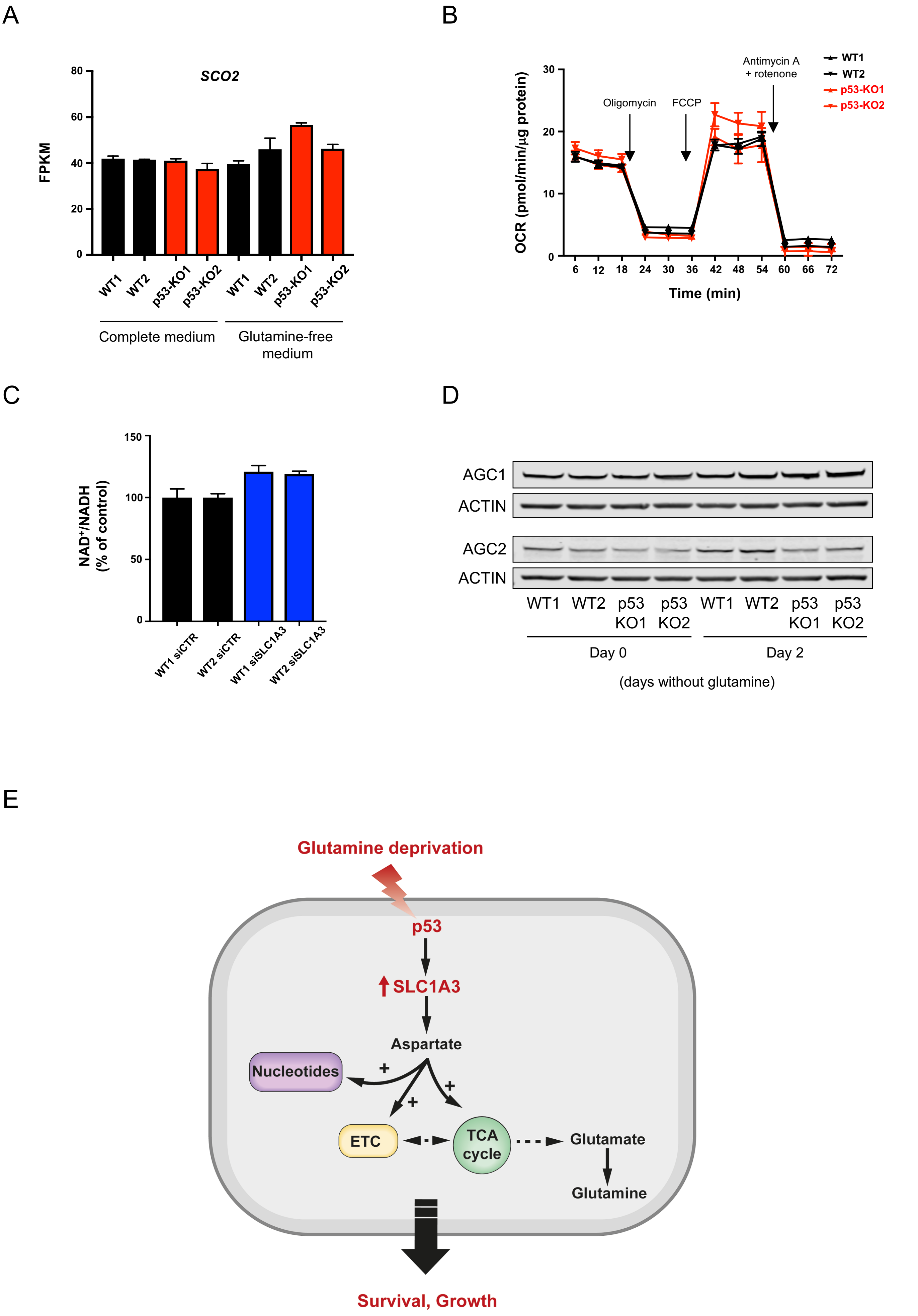

A
SCO2
FPKM
80
60
40
20
0
WT1
WT2
p53-KO1
p53-KO2
WT1
WT2
p53-KO1
p53-KO2
Complete medium
Glutamine-free medium
B
OCR (pmol/min/µg protein)
30
20
10
0
6
12
18
24
30
36
42
48
54
60
66
72
Time (min)
Oligomycin
FCCP
Antimycin A + rotenone
WT1
WT2
p53-KO1
p53-KO2
C
NAD⁺/NADH (% of control)
150
100
50
0
WT1 siCTR
WT2 siCTR
WT1 siSLC1A3
WT2 siSLC1A3
D
AGC1
ACTIN
AGC2
ACTIN
WT1
WT2
p53 KO1
p53 KO2
WT1
WT2
p53 KO1
p53 KO2
Day 0
Day 2
(days without glutamine)
E
Glutamine deprivation
p53
SLC1A3
Aspartate
Nucleotides
+
+
+
ETC
TCA cycle
Glutamate
Glutamine
Survival, Growth

Figure S7 (related to Figure 6): Impact of p53 and SLC1A3 on mitochondrial respiration.

A. Transcriptional expression of *SCO2* in HCT116 WT and p53-null clones grown for 2 days in complete medium or in glutamine-free medium. Data are presented as mean ± SEM (averages of triplicate wells).

B. Oxygen consumption rates (OCR) of HCT116 clones grown in complete media following the addition of mitochondrial inhibitors (oligomycin, FCCP, antimycin A & rotenone). Arrows indicate incubation of cells with the indicated compounds. Data are presented as mean ± SEM of one representative experiment (n=6 wells).

C. HCT116 WT cells transiently depleted of SLC1A3 using siRNA were grown for 2 days in glutamine-free medium and pulsed with U-[^{13}C]-aspartate for 16 hours. NAD^+/NADH ratio, expressed as a percentage of the control cells, was measured by LC-MS in cells fed glutamine-free medium. Data are presented as mean ± SEM of one representative experiment (averages of triplicate wells).

D. Western blots show AGC1 and AGC2 expression in HCT116 WT clones and p53-null clones grown for two days in glutamine-free medium.

Note, AGC2 and SLC1A3 (Figure 4B) were probed on the same membrane so the same ACTIN western blot is shown in Figure S7D and 4B.

E. Glutamine starvation induces a transient activation of p53 that is necessary to sustain the expression of the glutamate/aspartate transporter SLC1A3. SLC1A3 expression promotes aspartate utilization to support nucleotide synthesis, ETC and TCA cycle activity and *de novo* synthesis of glutamate and glutamine. SLC1A3 can therefore promote survival and growth of cancer cells under conditions of glutamine limitation.
